# Supplementary material for: The Mediator kinase module enhances polymerase activity to regulate transcriptional memory after heat stress in Arabidopsis
Source: EMBO J. 2024 Jan 16;43(3):6. doi: 10.1038/s44318-023-00024-x (PMC10897291; doi:10.1038/s44318-023-00024-x)
Supplement: Supplementary file 1 — Appendix with Figs S1, S2 [file 44318_2023_24_MOESM1_ESM.pdf]

Appendix for

Mediator kinase module enhances polymerase activity to regulate transcriptional memory after heat stress

Tim Crawford, Lara Siebler, Aleksandra Sulkowska, Bryan Nowack, Li Jiang, Yufeng Pan, Jörn Lämke, Christian Kappel, Isabel Bäurle

Table of Contents

Appendix Fig S1________________________________p. 2
Appendix Fig S2________________________________p. 3

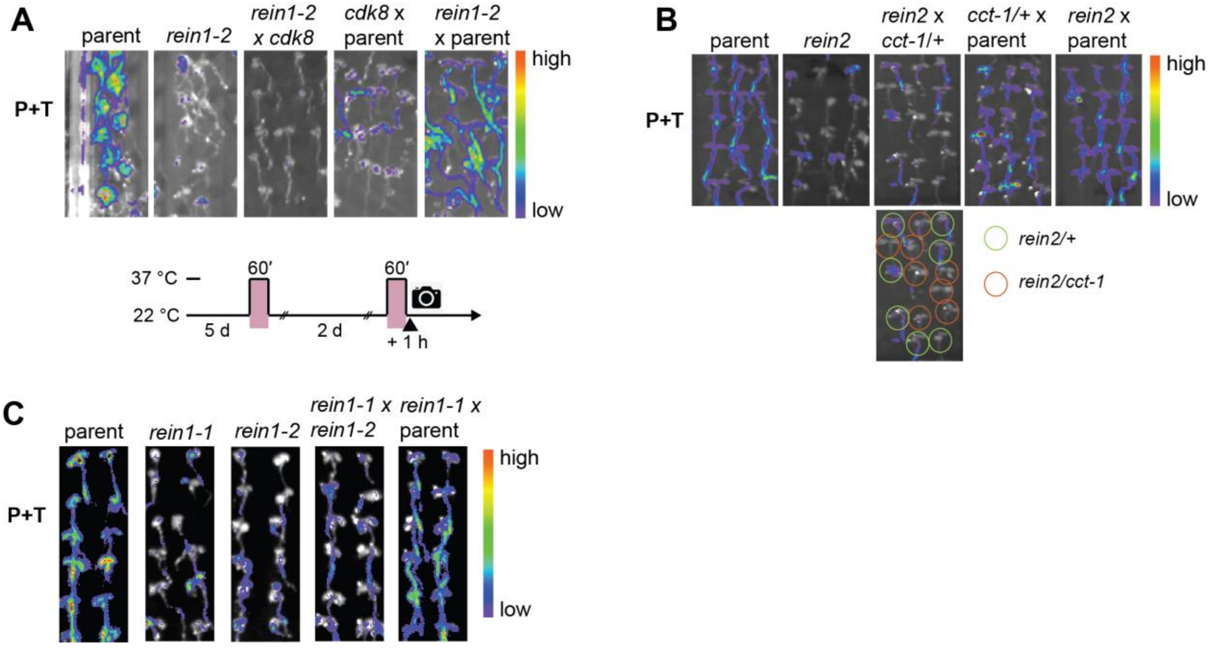

Appendix Fig S1. Allelism of *rein1-2* and *cdk8, rein2* and *med12, and rein1-1 and rein1-2* (cf. Fig 1)*.* A Allelism of *rein1-2* and *cdk8.* Bioluminescence in seedlings after repeated HS (P+T) was not complemented in the F1 progeny of *rein1-2* crossed with *cdk8*, but was complemented after crossing to the parental line. The schema below indicates the HS regime. Relative LUC (*pAPX2::LUC*) activity was converted to a false colour scale. **B** Allelism of *rein2* and *med12* (*cct-1*)*.* Bioluminescence in seedlings after repeated HS (P+T) was measured in the F1 progeny of *rein2* crossed to *cct-1/+*, as well as control crosses with the parental line. Due to the sterility of homozygous *cct-1* mutants, heterozygous individuals were used for crosses; after imaging, individual seedlings were genotyped. *Rein2/cct-1* seedlings failed to complement the LUC phenotype while *rein2/+* seedlings showed complementation. **C** Allelism of *rein1-1* and *rein1-2.* Bioluminescence in seedlings after repeated HS (P+T) was not complemented in the F1 progeny of *rein1-1* crossed with *rein1-2*, but was complemented after crossing to the parental line.

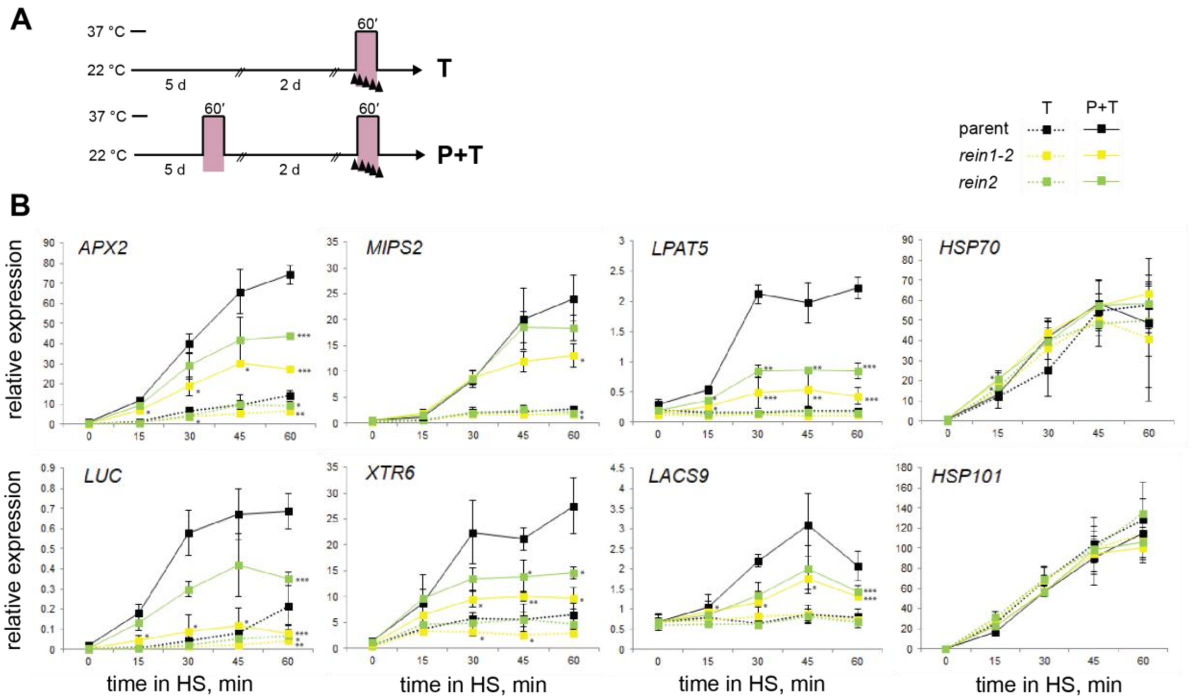

Appendix Fig S2. Kinetics of gene hyper-induction are altered in *rein* mutants (cf Fig 3). **A** Treatment schema for the Type II HS memory assay. T, HS on d 7 only (triggered); P+T, HS on both d 5 and d 7 (primed + triggered). Naïve or primed seedlings were sampled immediately prior to and at 15-minute intervals during the triggering HS, up to 60 min (arrowheads). **B** Seedlings were treated as indicated and relative transcript levels of six type II memory genes (*APX2*, *pAPX2*::*LUC*, *MIPS2*, *LPAT5*, *XTR6*, *LACS9*) and two HS-induced non-memory genes (*HSP70*, *HSP101*) were measured by qRT-PCR and normalized to the expression of *At4g26410*. Data are mean ± SEM of three independent biological replicates. Asterisks denote significant difference of the *rein1-2* or *rein2* mutant to the parental line (*, p < 0.05; **, p < 0.005; ***, p < 0.0005; unpaired, two-tailed t-test).
