# Supplementary figures and images for: The Mediator kinase module enhances polymerase activity to regulate transcriptional memory after heat stress in Arabidopsis
Source: EMBO J. 2024 Jan 16;43(3):6. doi: 10.1038/s44318-023-00024-x (PMC10897291; doi:10.1038/s44318-023-00024-x)

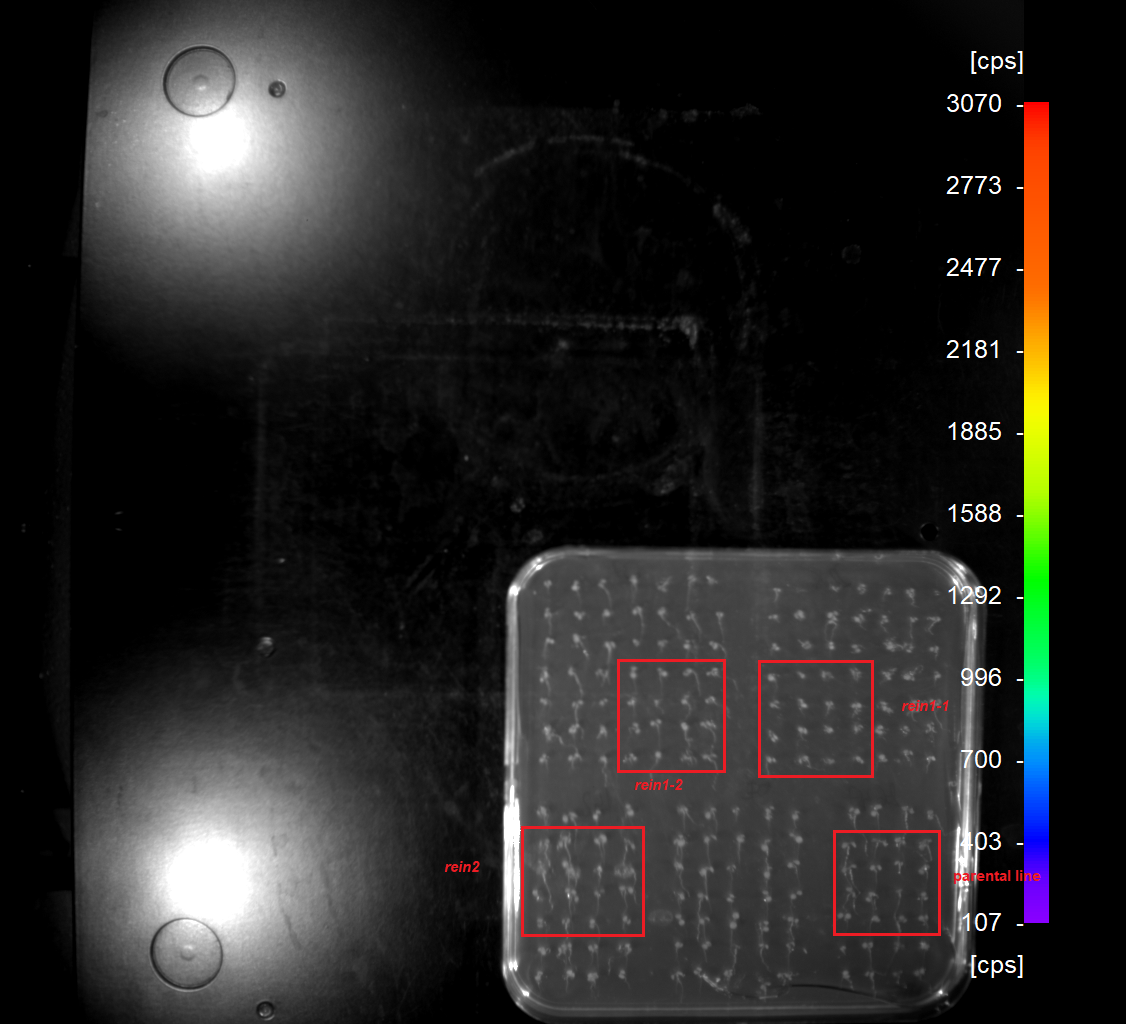

Supplement: Supplementary file 5 — Source Data Fig. 1 [file 44318_2023_24_MOESM5_ESM.zip › Figure 1/1B/Fig 1B rein-mutants-luciferase-assay-NHS_annotated.png]

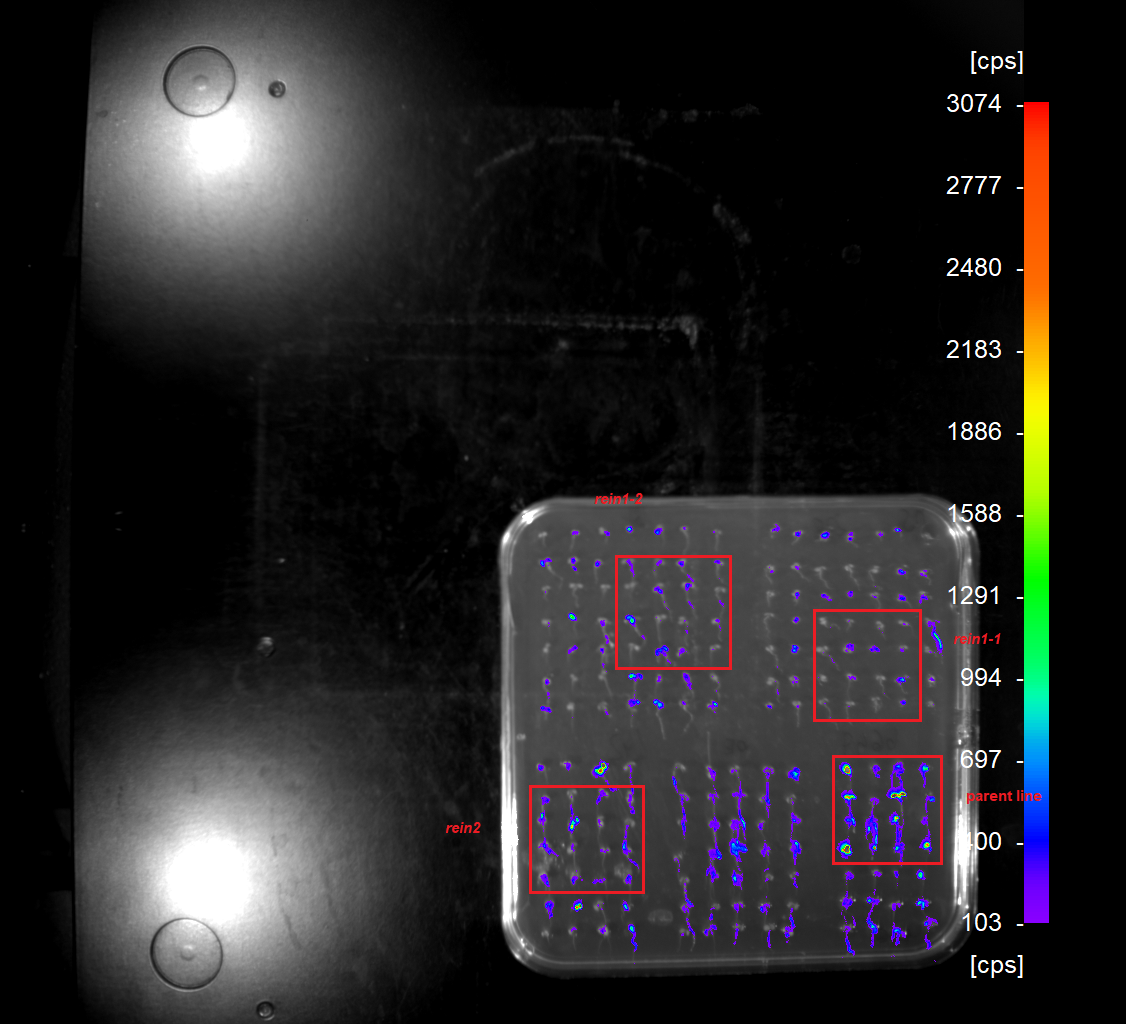

Supplement: Supplementary file 5 — Source Data Fig. 1 [file 44318_2023_24_MOESM5_ESM.zip › Figure 1/1B/Fig 1B rein-mutants-luciferase-assay-P+T_annotated.png]

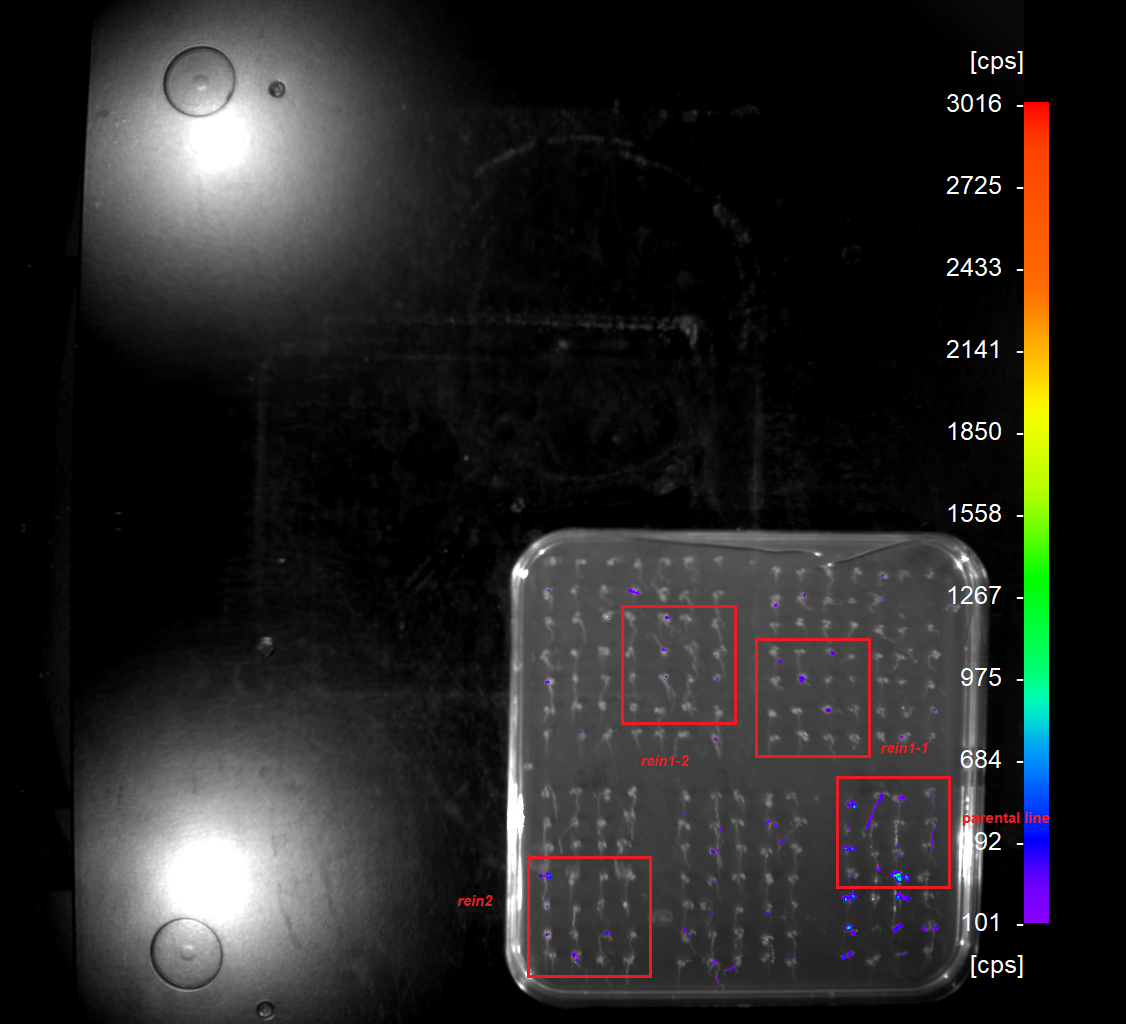

Supplement: Supplementary file 5 — Source Data Fig. 1 [file 44318_2023_24_MOESM5_ESM.zip › Figure 1/1B/Fig 1B rein-mutants-luciferase-assay-P_annotated.png]

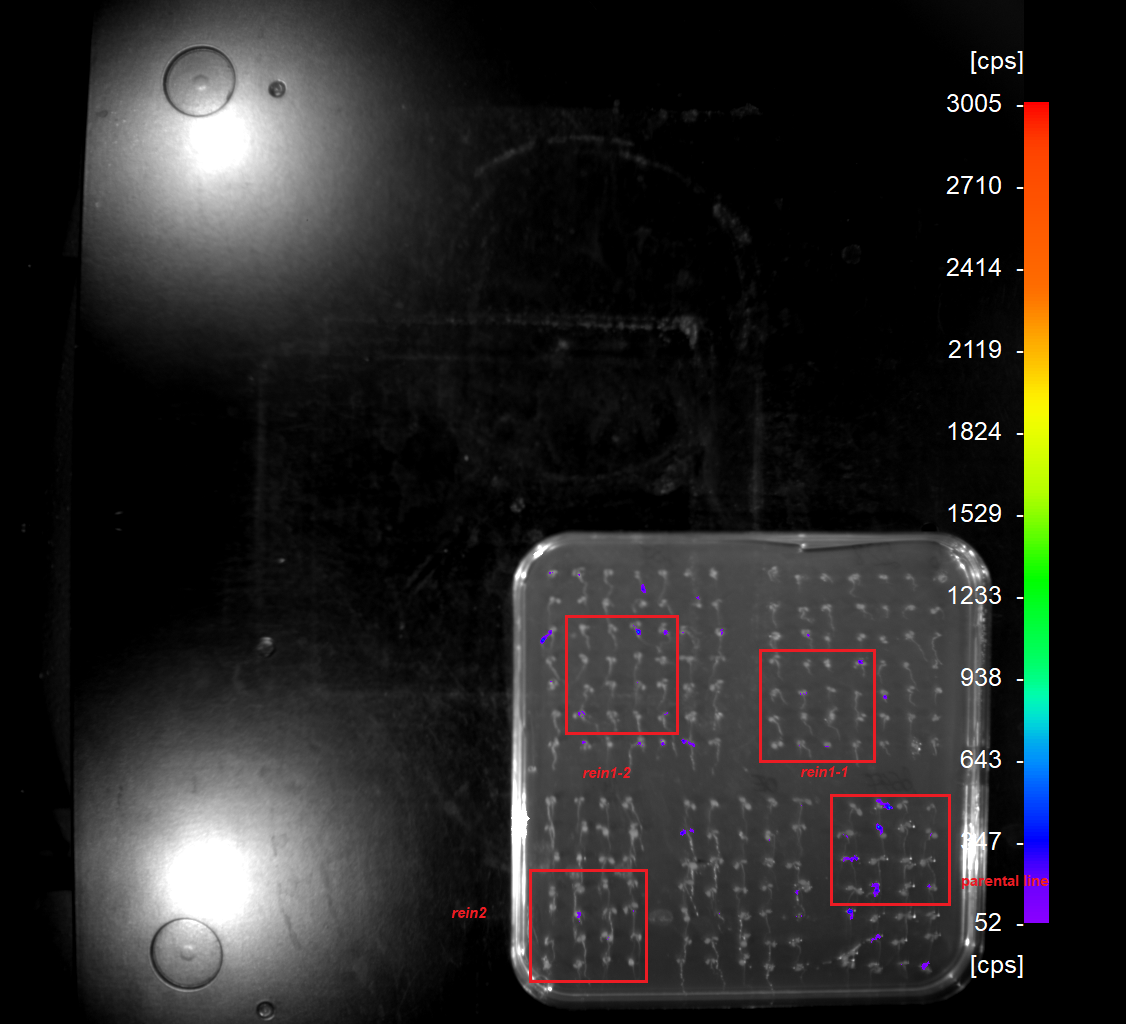

Supplement: Supplementary file 5 — Source Data Fig. 1 [file 44318_2023_24_MOESM5_ESM.zip › Figure 1/1B/Fig 1B rein-mutants-luciferase-assay-T_annotated.png]

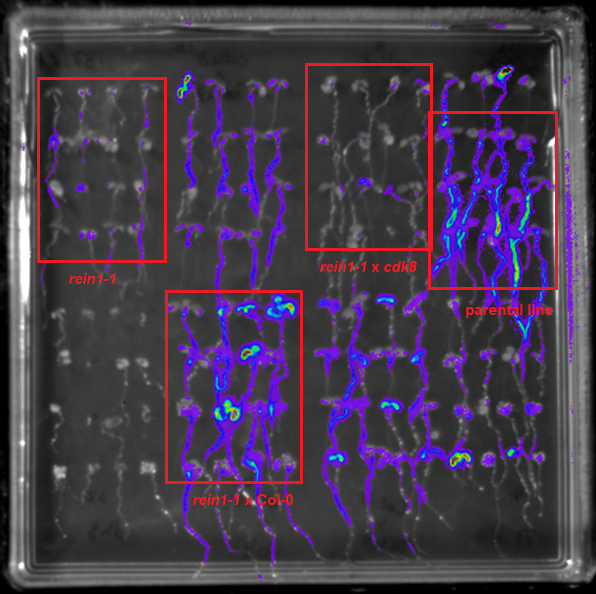

Supplement: Supplementary file 5 — Source Data Fig. 1 [file 44318_2023_24_MOESM5_ESM.zip › Figure 1/1D/Fig 1D rein1-1-crossings-P+T_annotated.png]

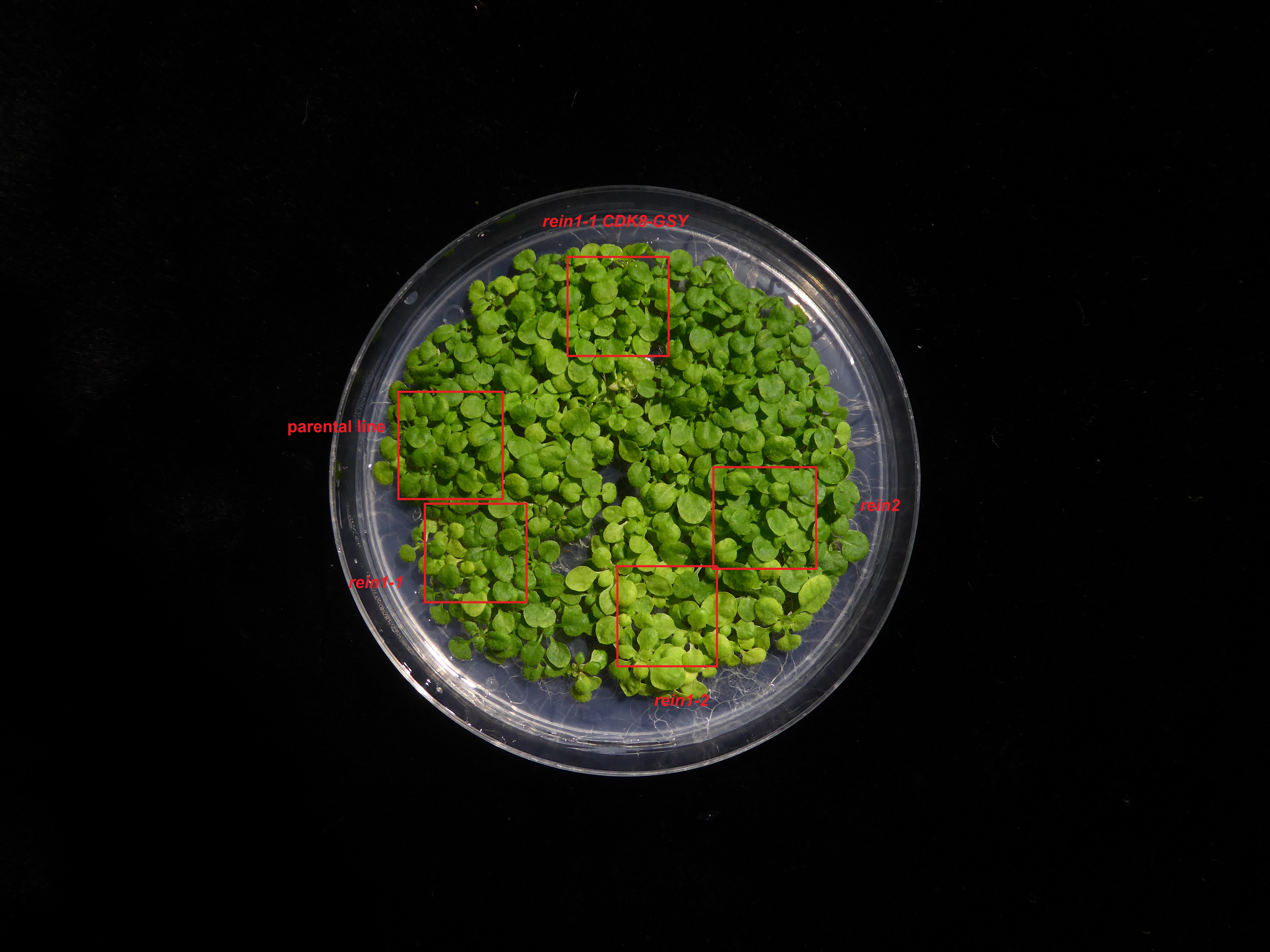

Supplement: Supplementary file 6 — Source Data Fig. 2 [file 44318_2023_24_MOESM6_ESM.zip › Figure 2/2B/Fig 2B rein_maTT_ACC-only_annotated.jpg]

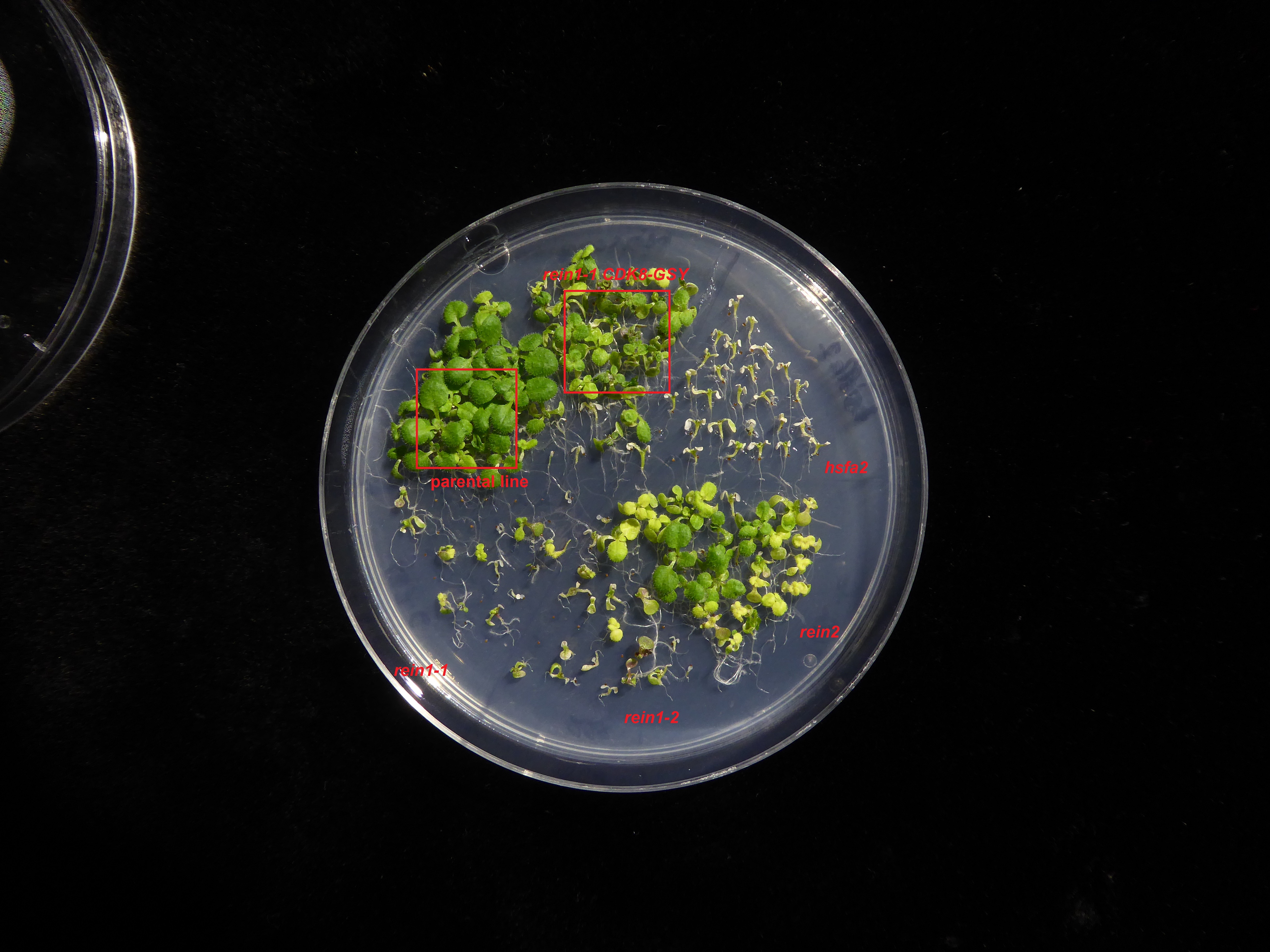

Supplement: Supplementary file 6 — Source Data Fig. 2 [file 44318_2023_24_MOESM6_ESM.zip › Figure 2/2B/Fig 2B rein_maTT_HS_d+3_70'_annotated n1.jpg]

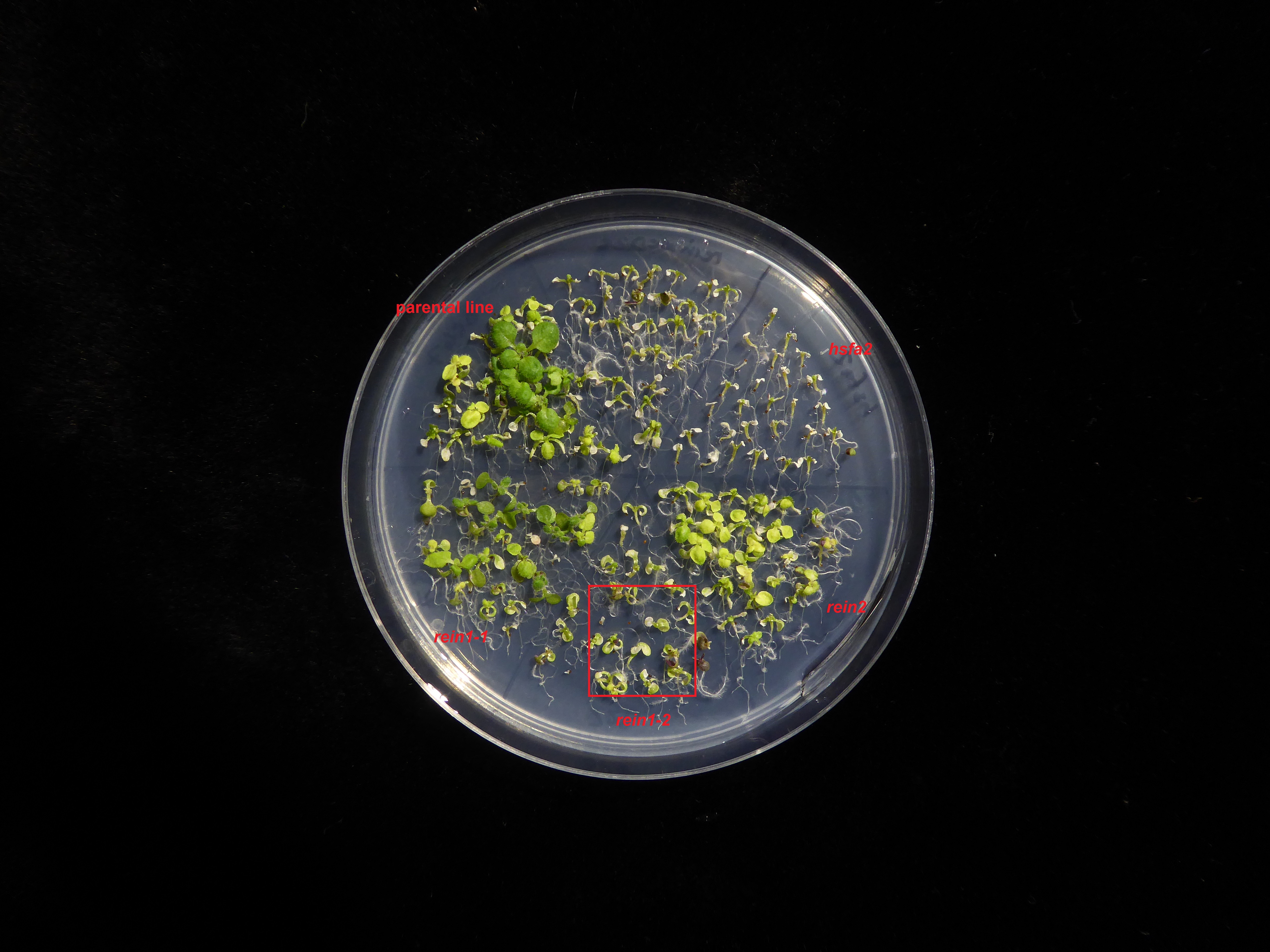

Supplement: Supplementary file 6 — Source Data Fig. 2 [file 44318_2023_24_MOESM6_ESM.zip › Figure 2/2B/Fig 2B rein_maTT_HS_d+3_70'_annotated n2.jpg]

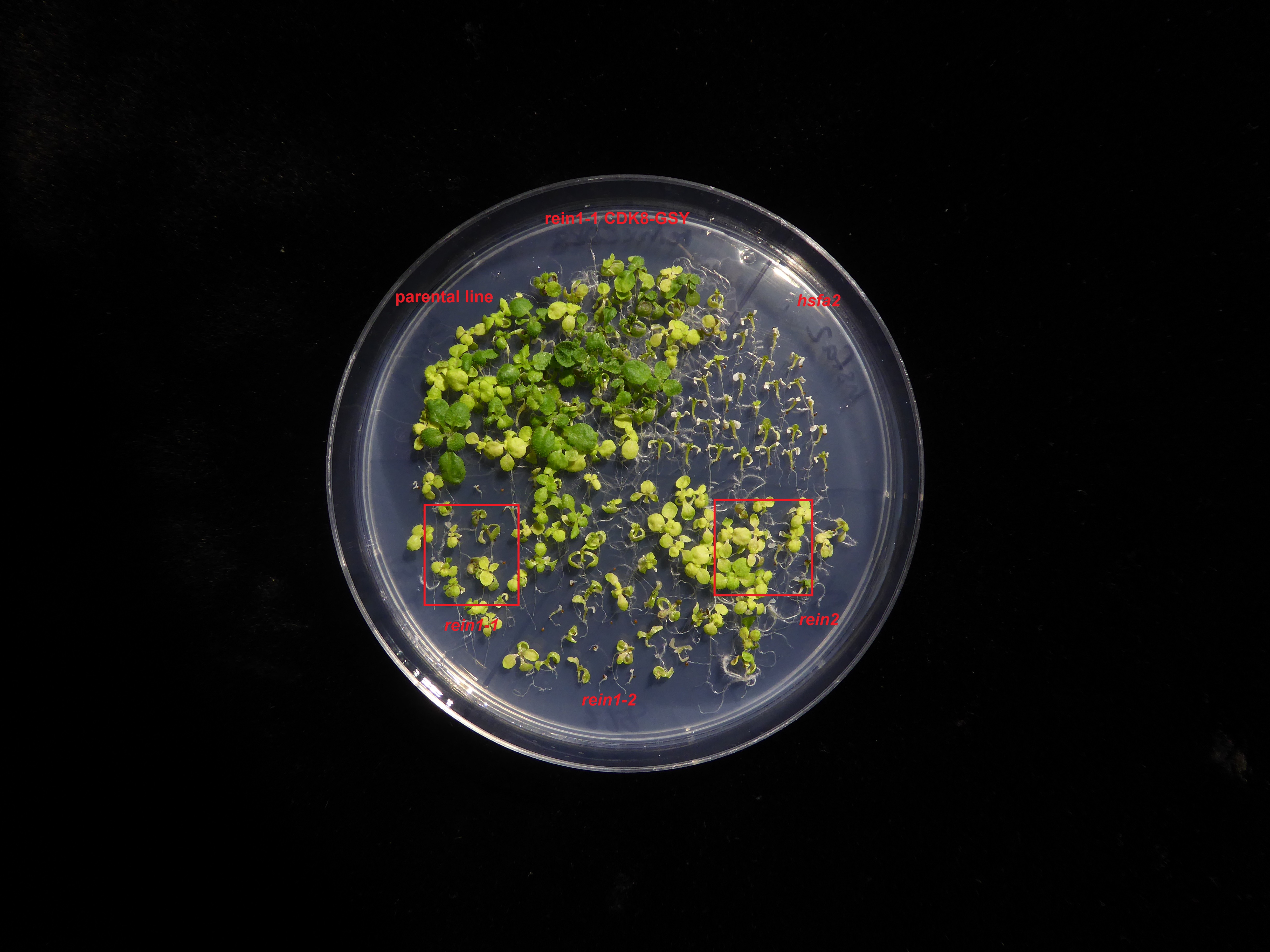

Supplement: Supplementary file 6 — Source Data Fig. 2 [file 44318_2023_24_MOESM6_ESM.zip › Figure 2/2B/Fig 2B rein_maTT_HS_d+3_70'_annotated n3.jpg]

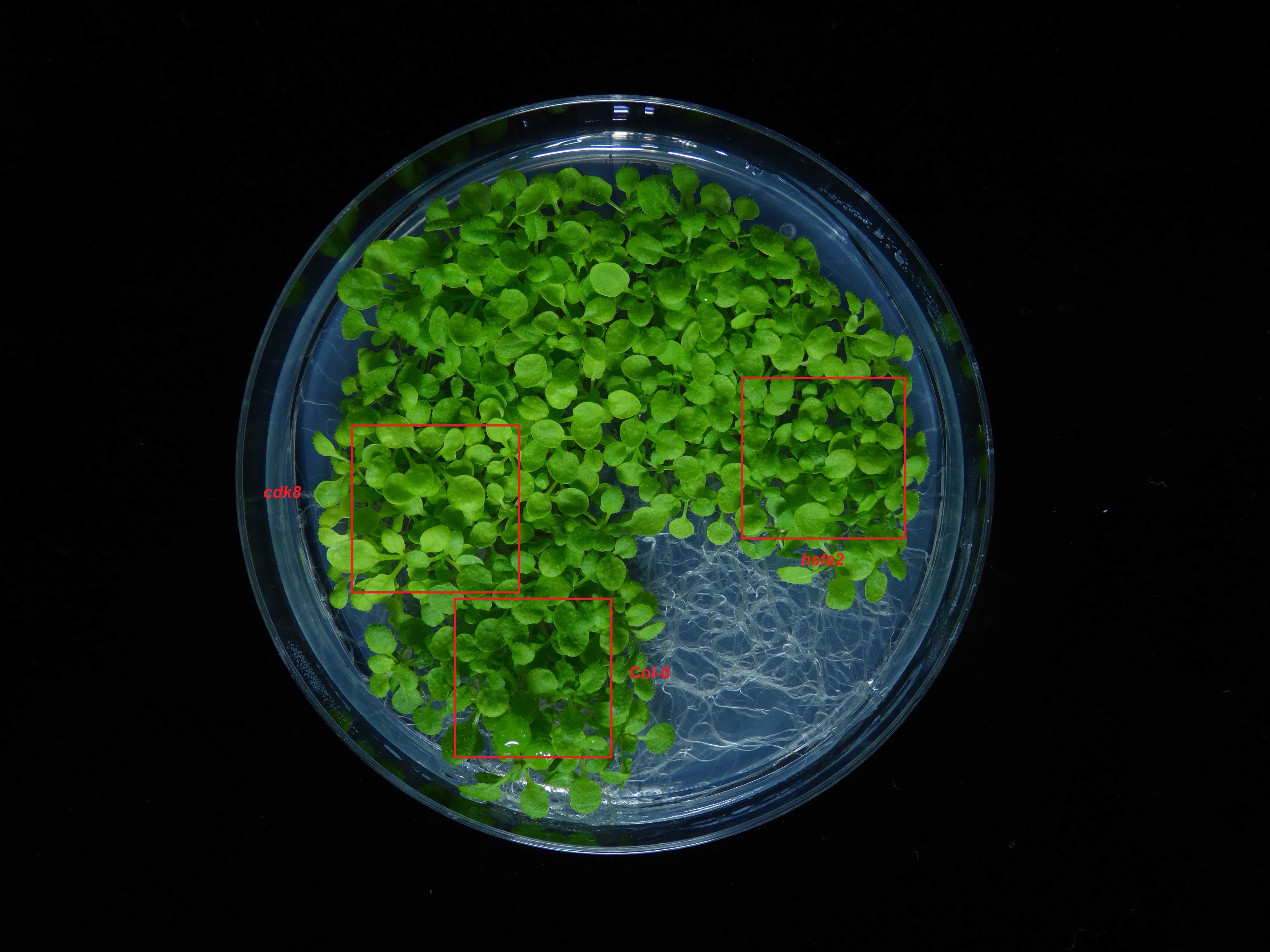

Supplement: Supplementary file 6 — Source Data Fig. 2 [file 44318_2023_24_MOESM6_ESM.zip › Figure 2/2C/Fig 2C cdk8_maTT_ACC_annotated.jpg]

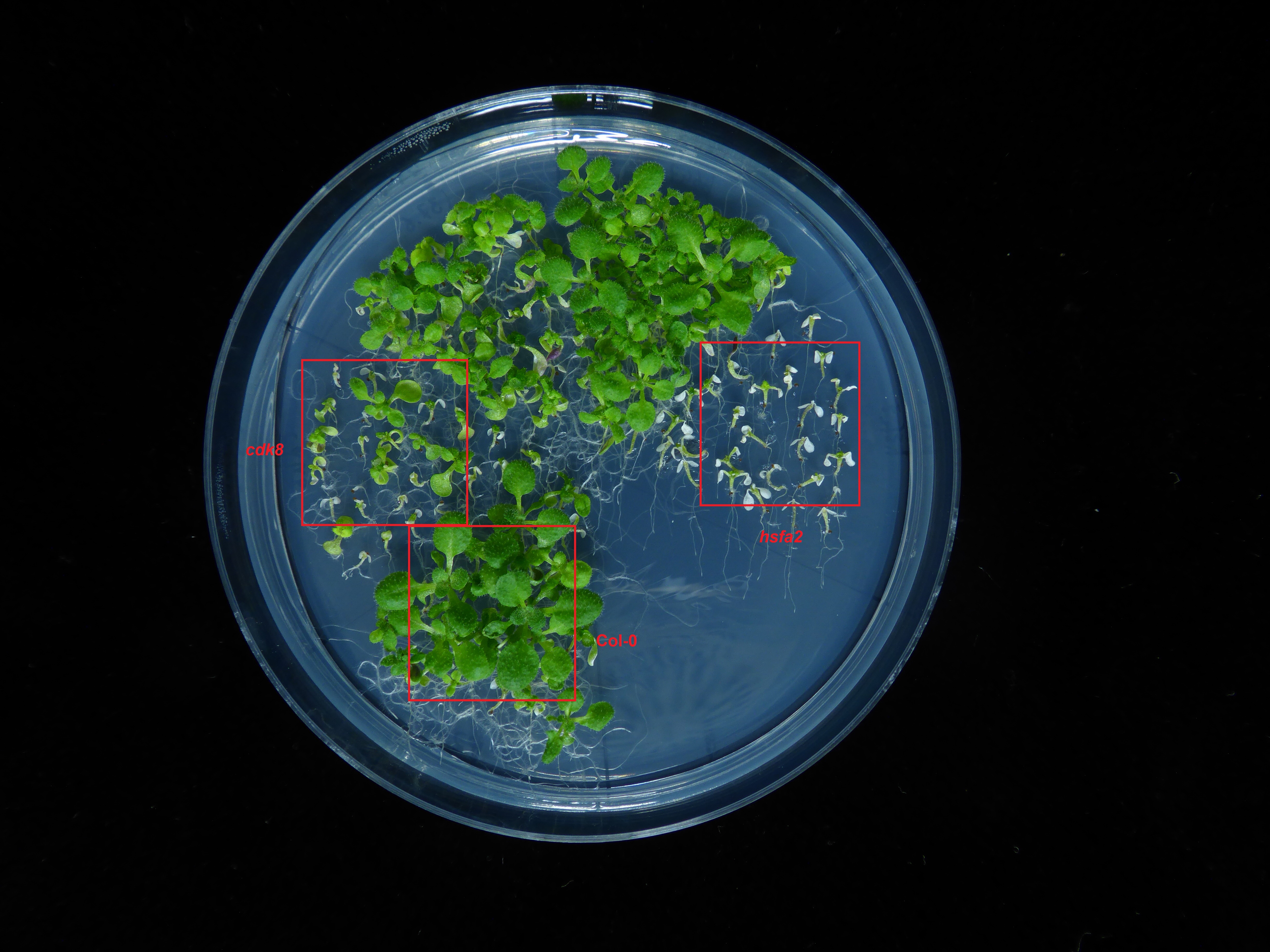

Supplement: Supplementary file 6 — Source Data Fig. 2 [file 44318_2023_24_MOESM6_ESM.zip › Figure 2/2C/Fig 2C cdk8_maTT_HS_d+3_70'_annotated.jpg]

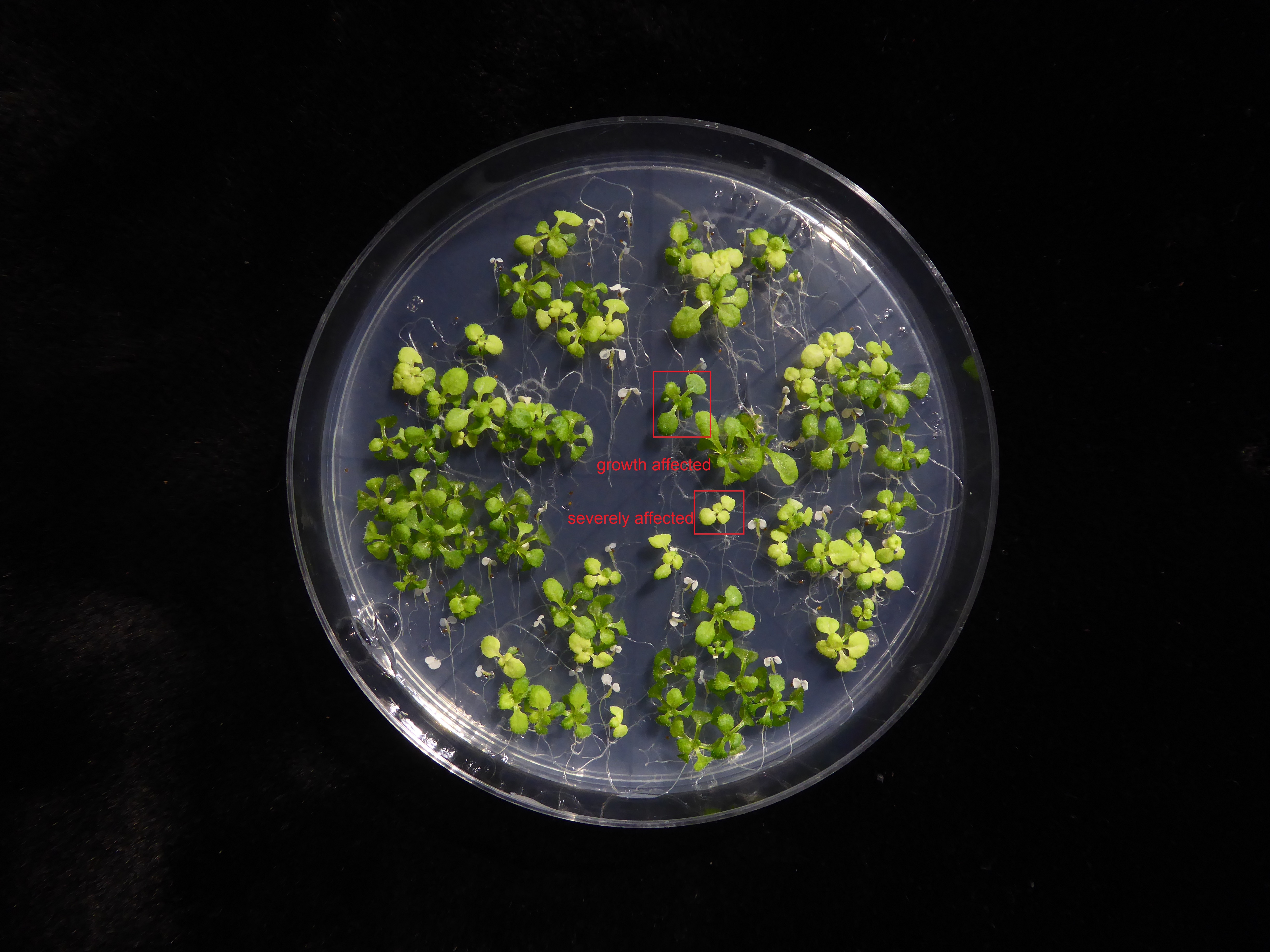

Supplement: Supplementary file 6 — Source Data Fig. 2 [file 44318_2023_24_MOESM6_ESM.zip › Figure 2/2D/Fig 2D phenotypes key (affected + severely affected).jpg]

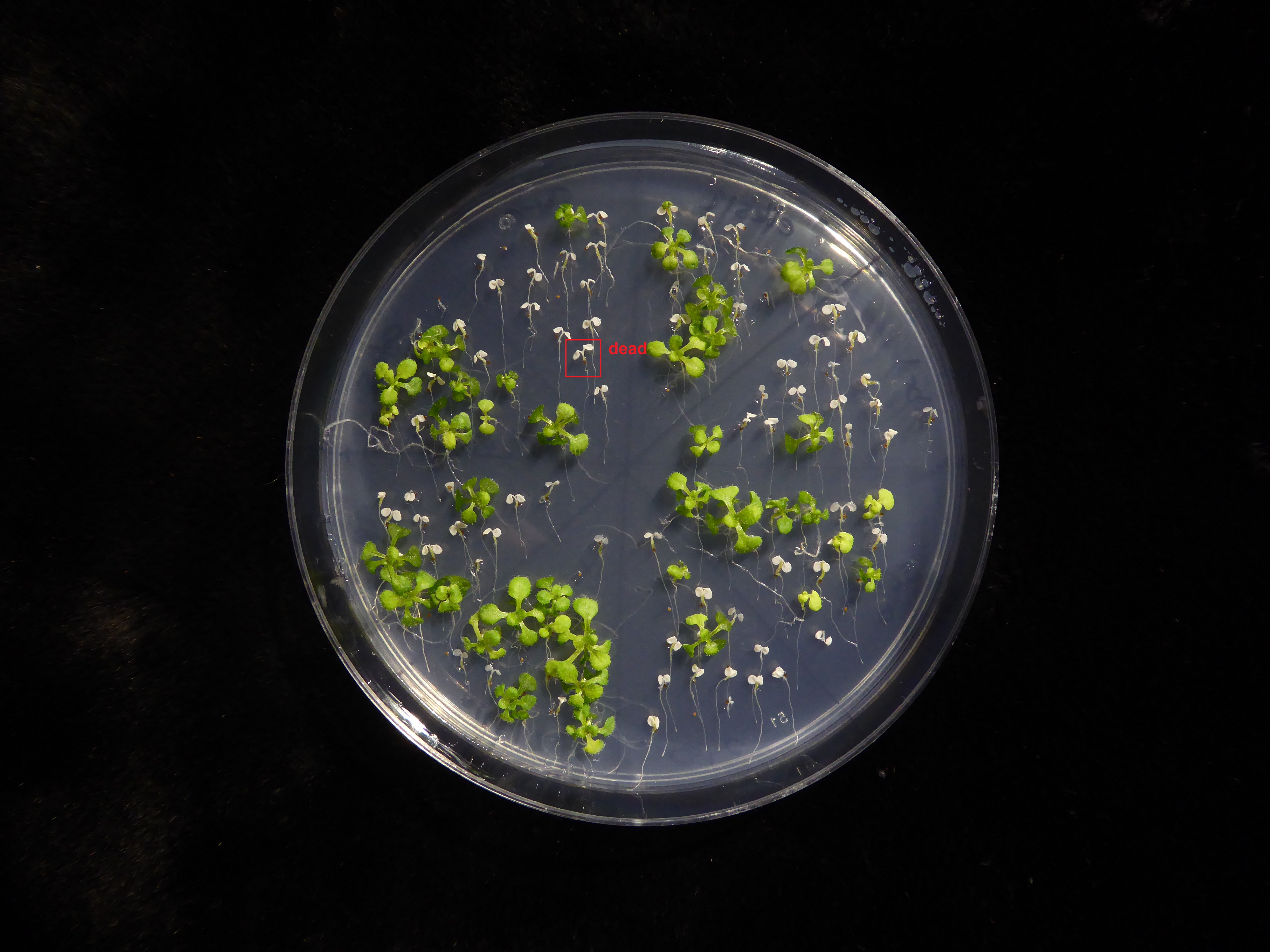

Supplement: Supplementary file 6 — Source Data Fig. 2 [file 44318_2023_24_MOESM6_ESM.zip › Figure 2/2D/Fig 2D phenotypes key (dead).jpg]

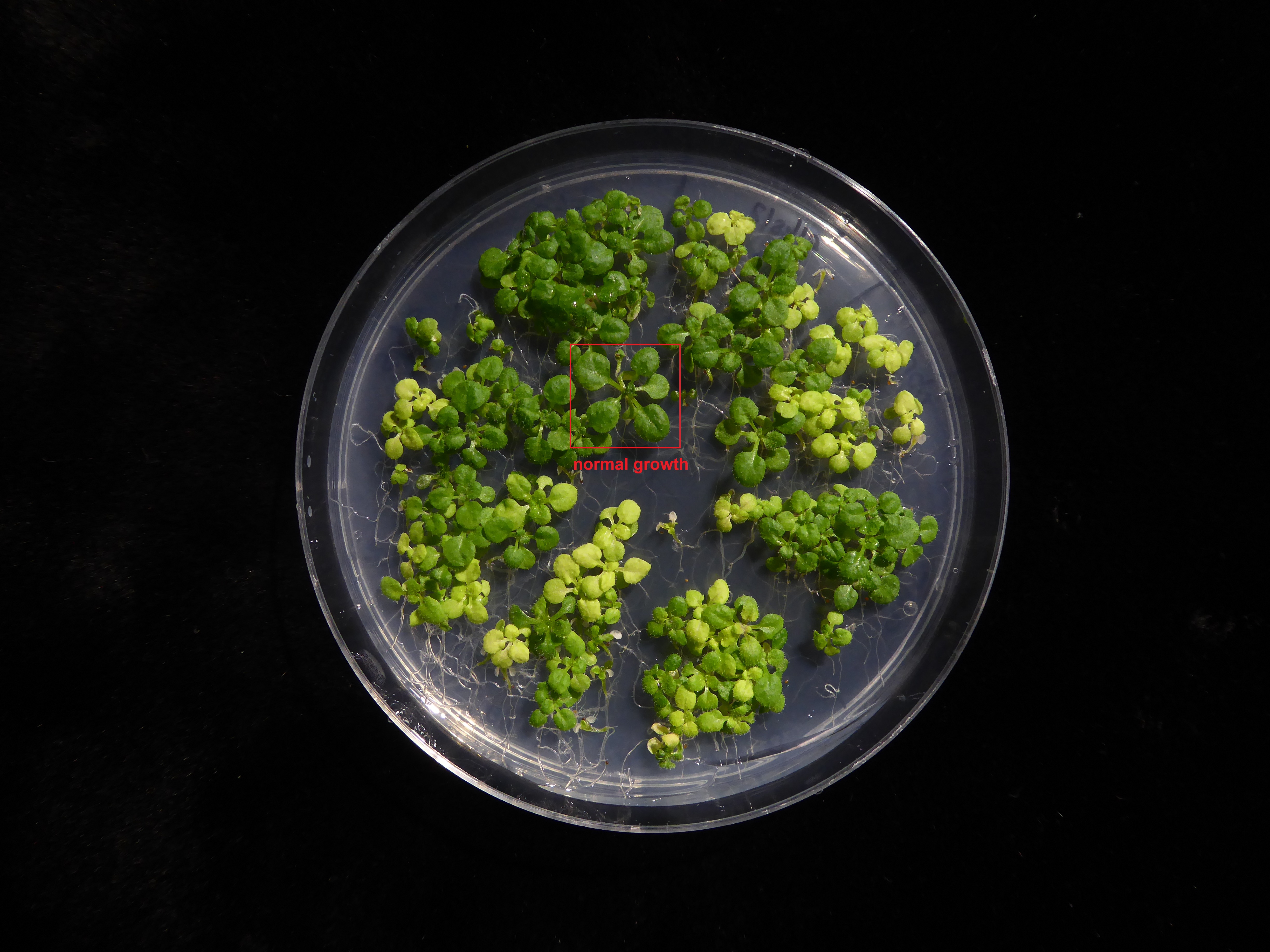

Supplement: Supplementary file 6 — Source Data Fig. 2 [file 44318_2023_24_MOESM6_ESM.zip › Figure 2/2D/Fig 2D phenotypes key (normal).jpg]

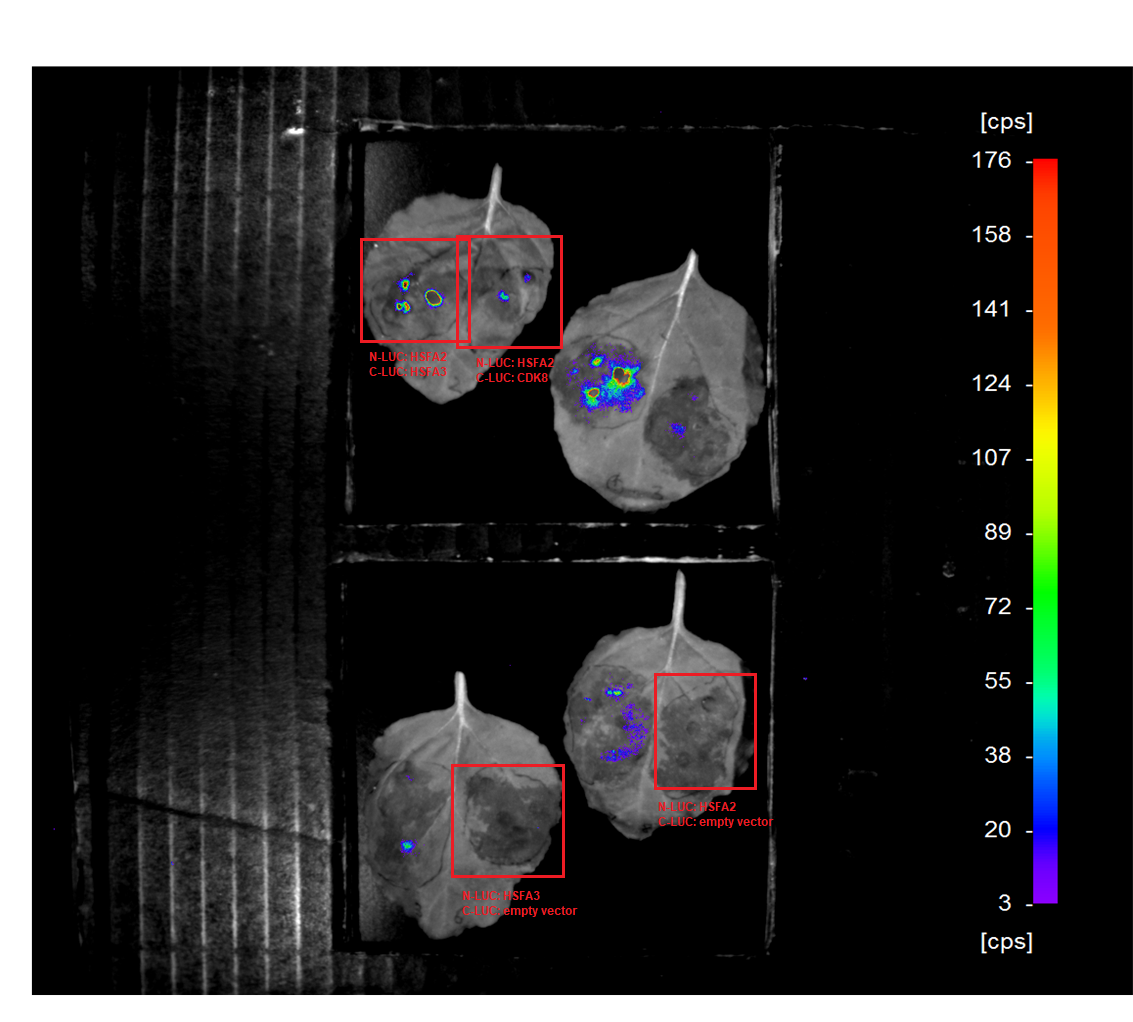

Supplement: Supplementary file 9 — Source Data Fig. 6 [file 44318_2023_24_MOESM9_ESM.zip › Figure 6/6A/Fig 6A split-luc-A2-A3_CDK8-A2_rep1.png]

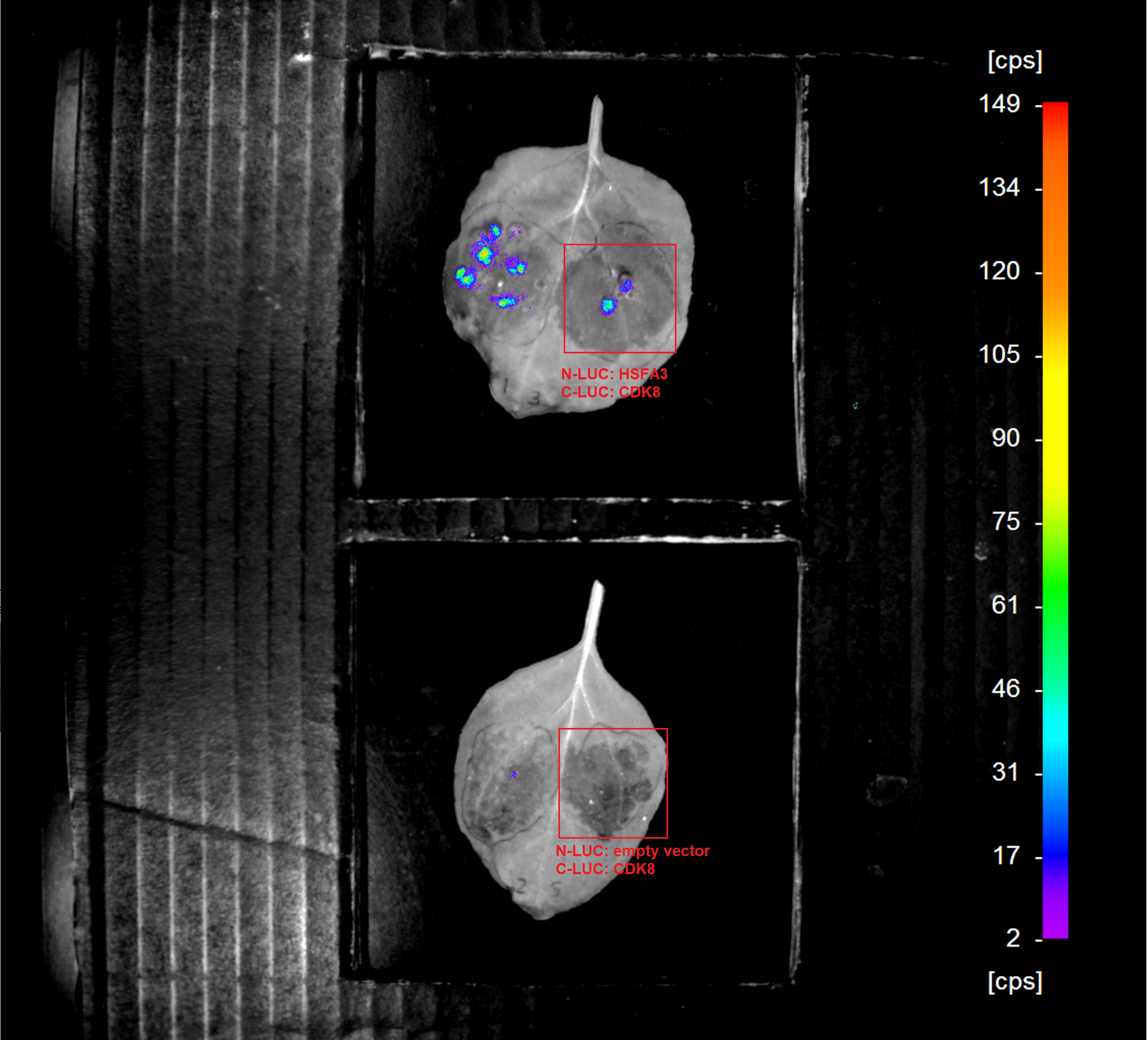

Supplement: Supplementary file 9 — Source Data Fig. 6 [file 44318_2023_24_MOESM9_ESM.zip › Figure 6/6A/Fig 6A split-luc-CDK8-A3_rep1.png]

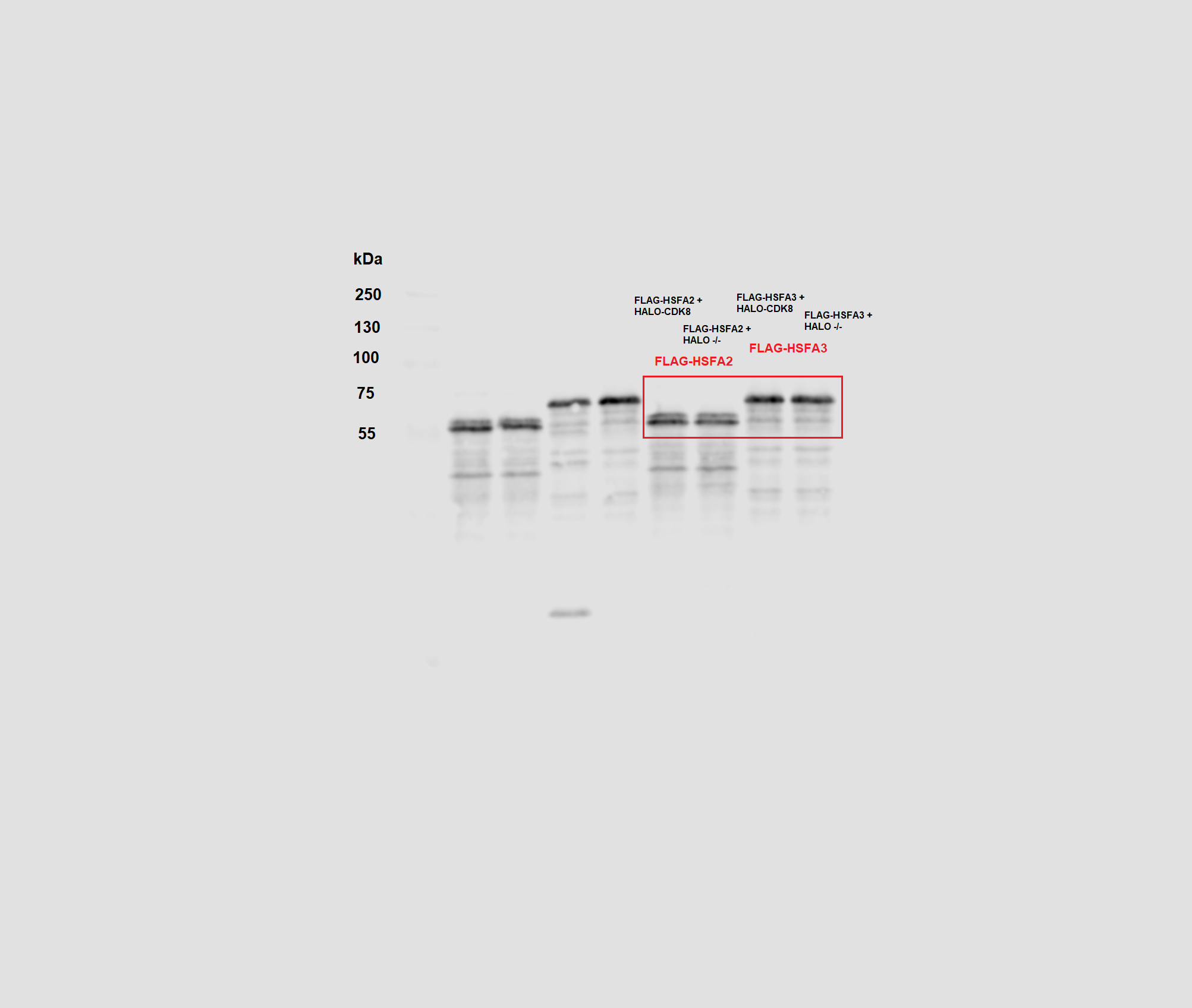

Supplement: Supplementary file 9 — Source Data Fig. 6 [file 44318_2023_24_MOESM9_ESM.zip › Figure 6/6B/Fig 6B in-vitro-co-ip-input-FLAG.tif]

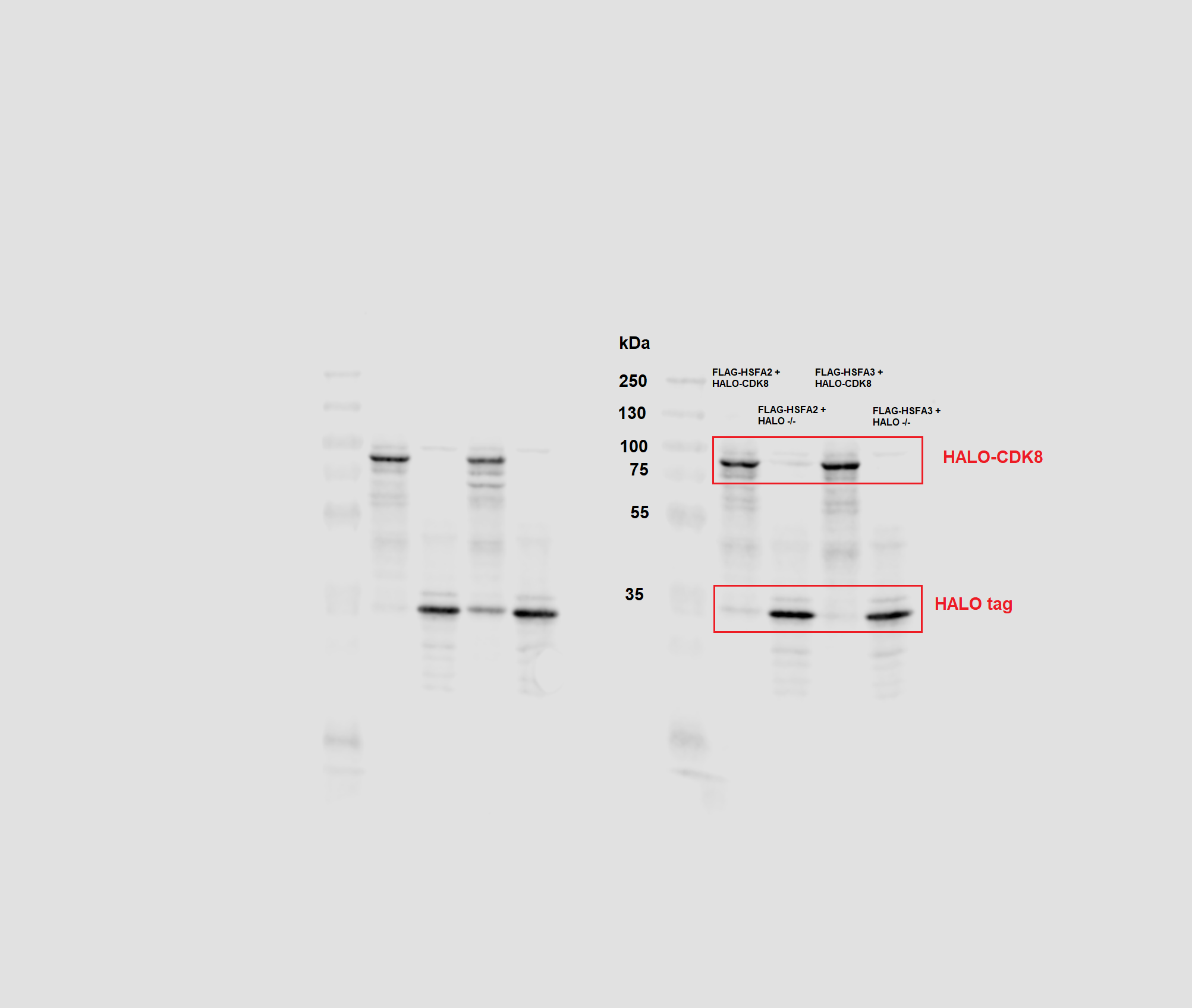

Supplement: Supplementary file 9 — Source Data Fig. 6 [file 44318_2023_24_MOESM9_ESM.zip › Figure 6/6B/Fig 6B in-vitro-co-ip-input-HALO.tif]

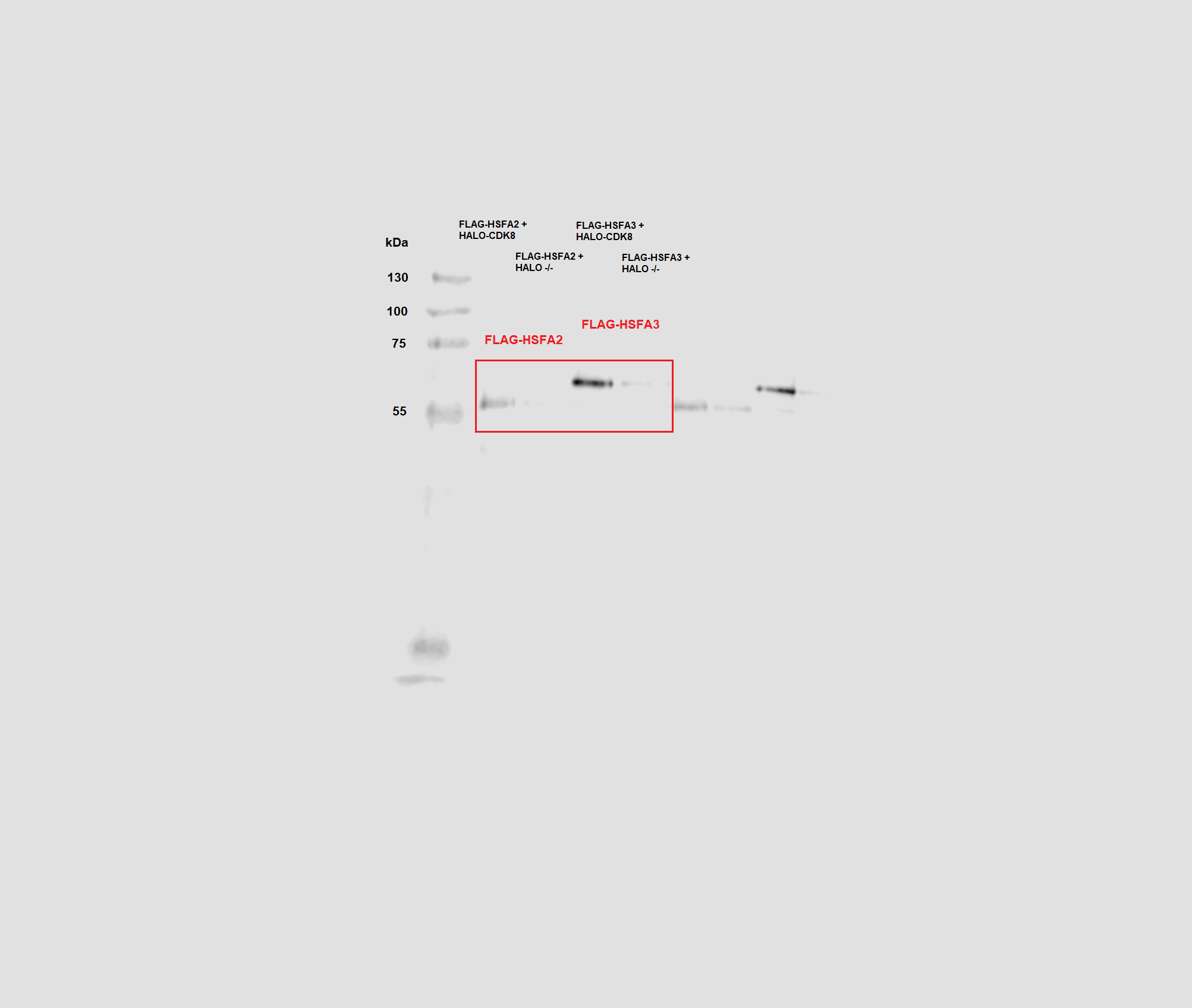

Supplement: Supplementary file 9 — Source Data Fig. 6 [file 44318_2023_24_MOESM9_ESM.zip › Figure 6/6B/Fig 6B in-vitro-co-ip-ip-halo-FLAG.tif]

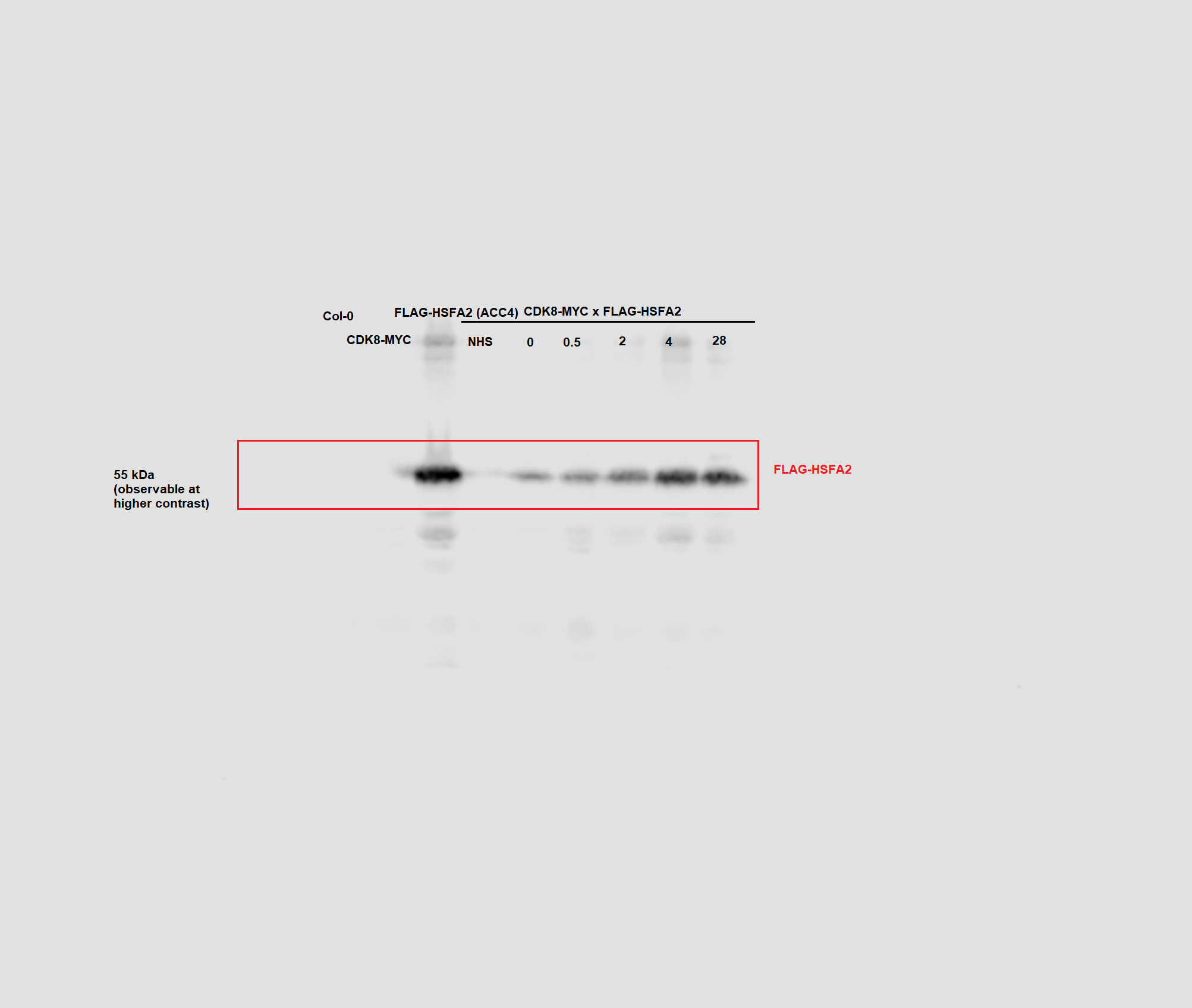

Supplement: Supplementary file 9 — Source Data Fig. 6 [file 44318_2023_24_MOESM9_ESM.zip › Figure 6/6C/Fig 6C in-vivo-co-ip-input-flag.tif]

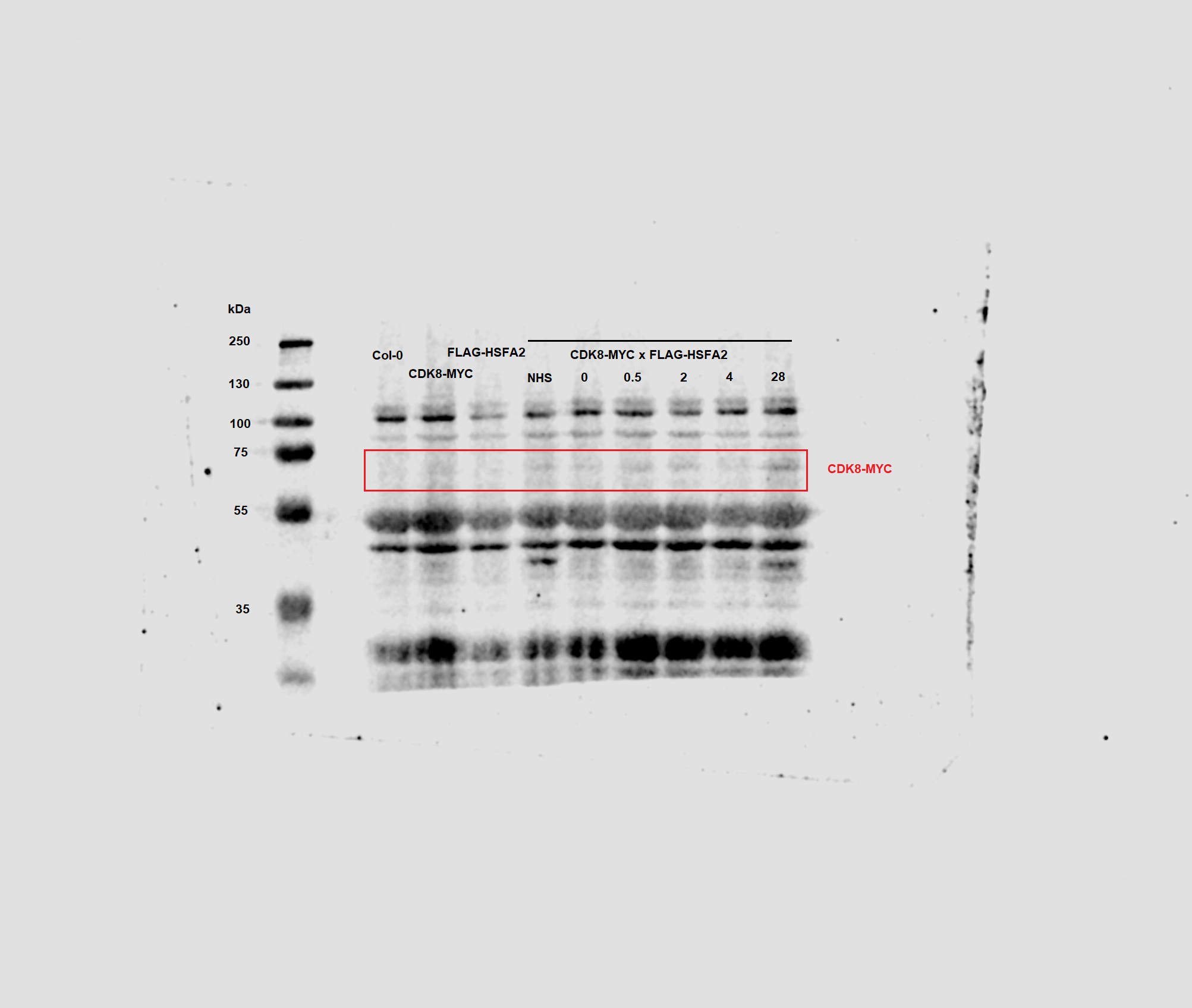

Supplement: Supplementary file 9 — Source Data Fig. 6 [file 44318_2023_24_MOESM9_ESM.zip › Figure 6/6C/Fig 6C in-vivo-co-ip-input-myc.tif]

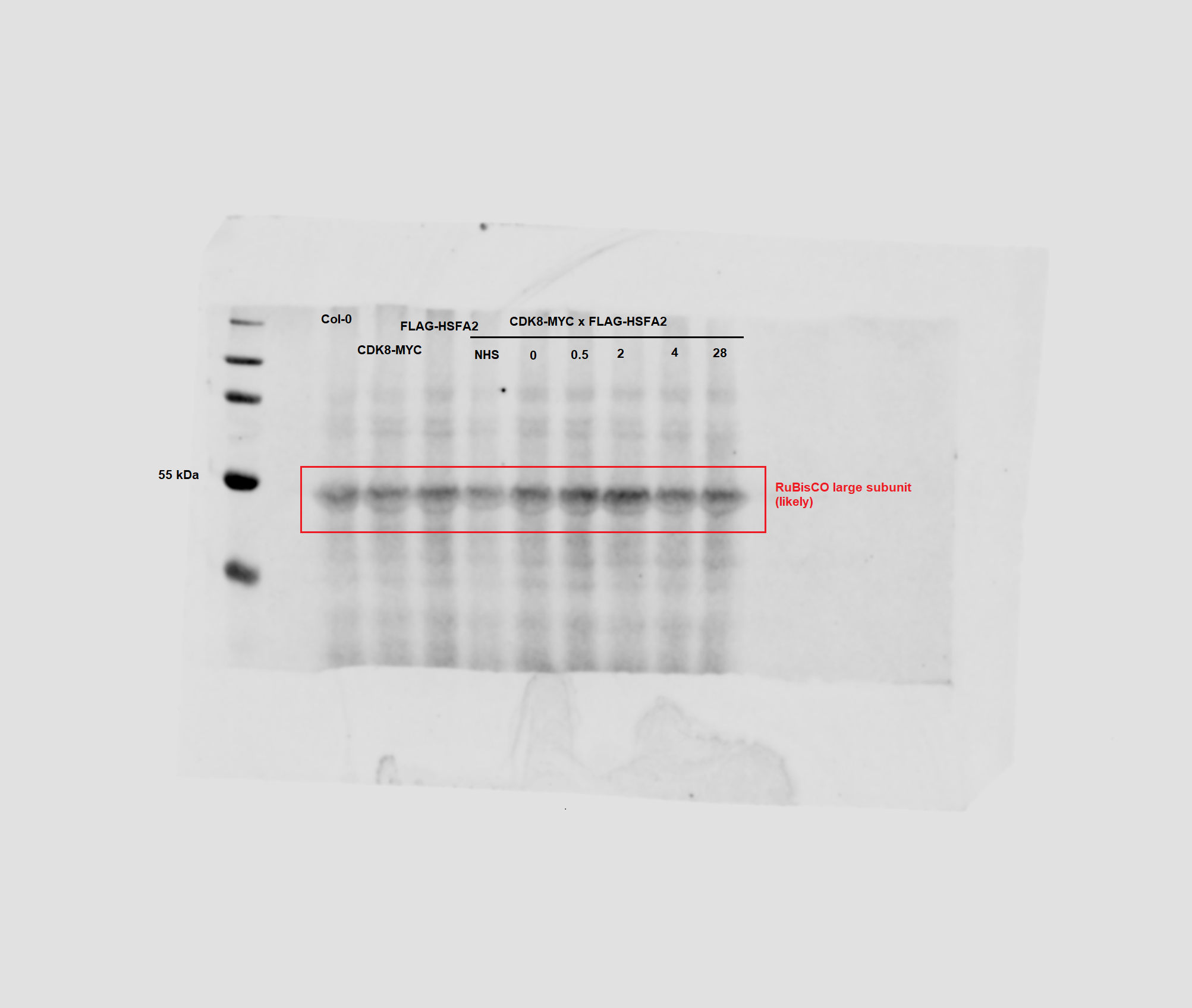

Supplement: Supplementary file 9 — Source Data Fig. 6 [file 44318_2023_24_MOESM9_ESM.zip › Figure 6/6C/Fig 6C in-vivo-co-ip-input-revert-stain.tif]

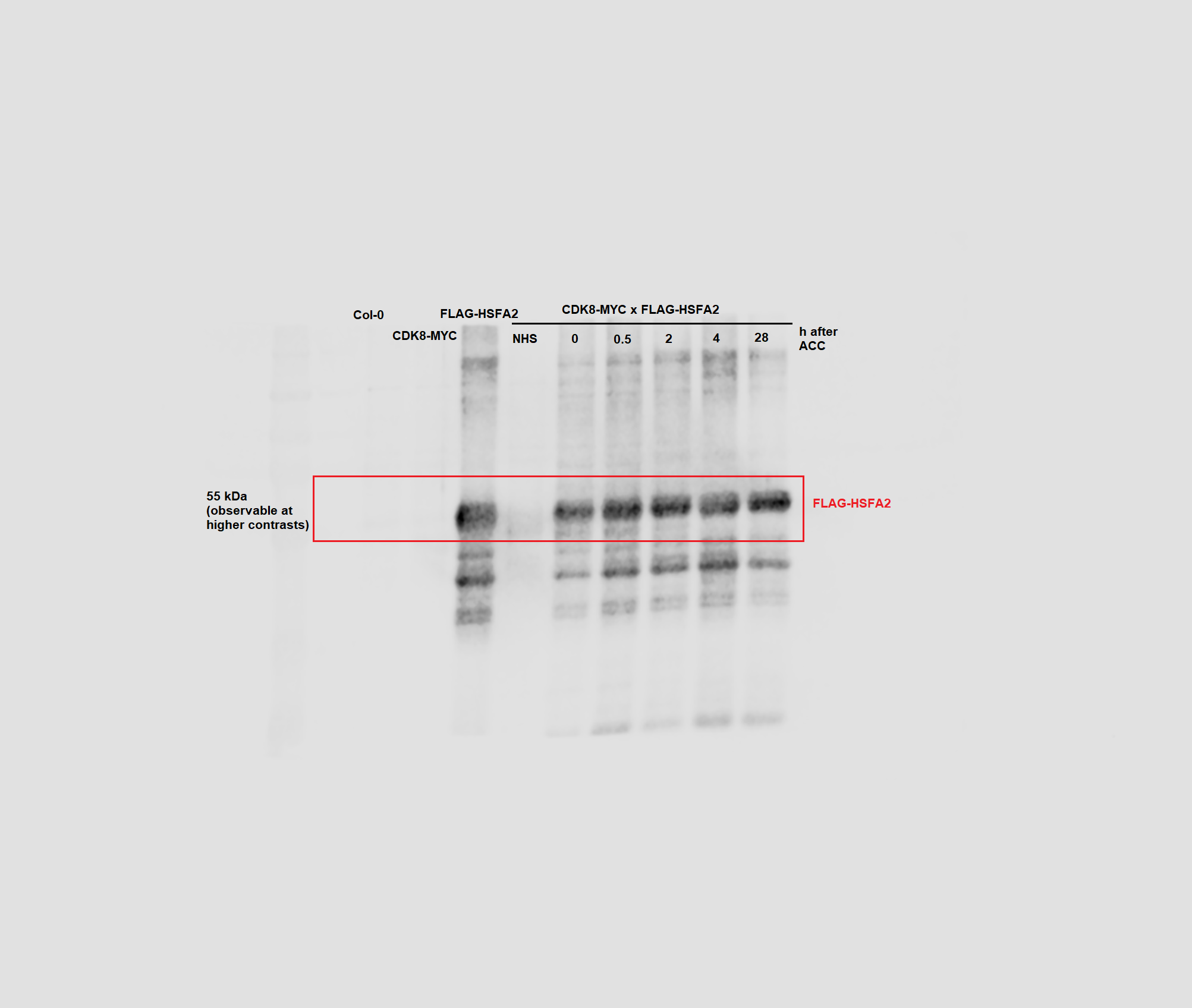

Supplement: Supplementary file 9 — Source Data Fig. 6 [file 44318_2023_24_MOESM9_ESM.zip › Figure 6/6C/Fig 6C in-vivo-co-ip-ip-flag-flag.tif]

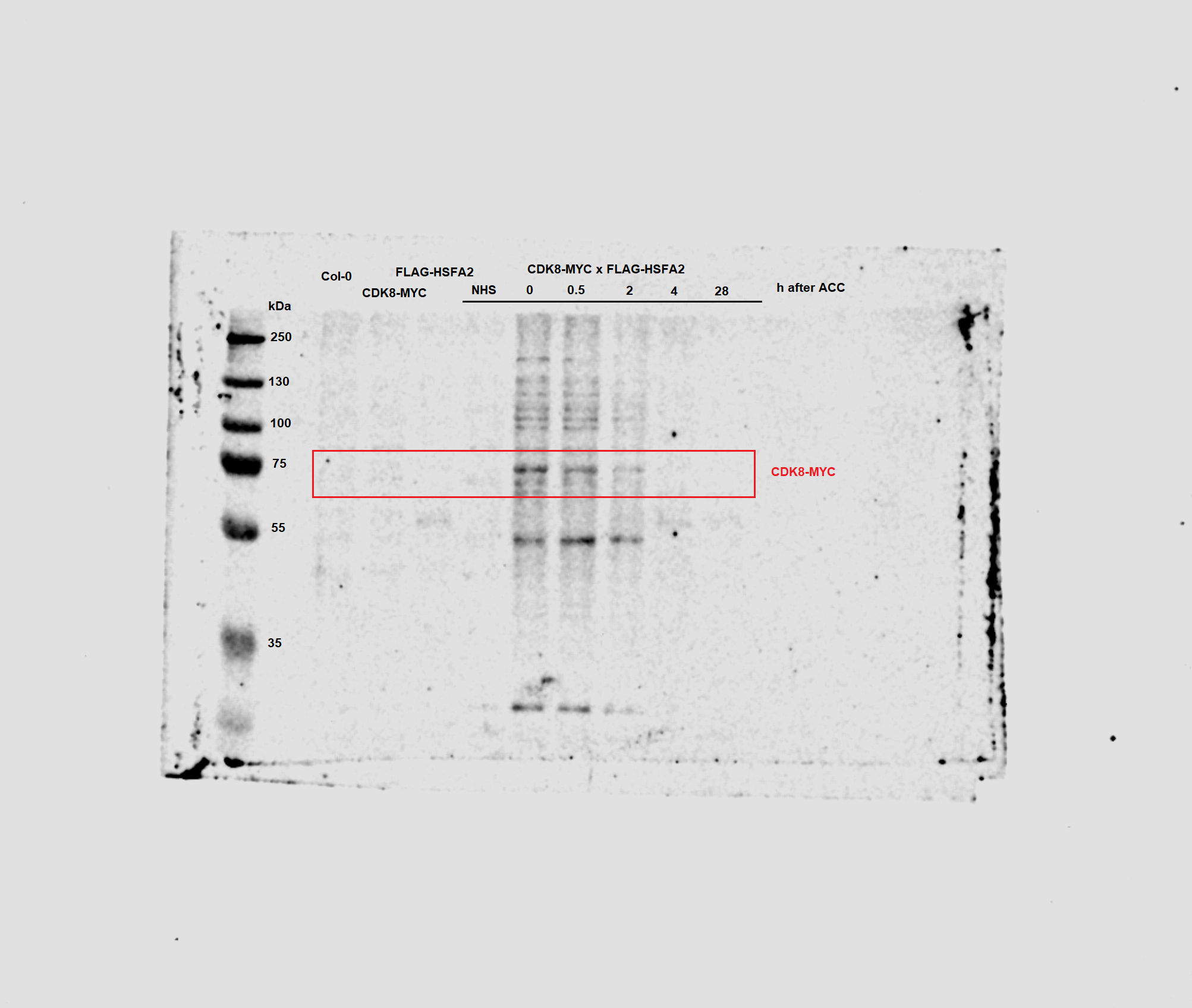

Supplement: Supplementary file 9 — Source Data Fig. 6 [file 44318_2023_24_MOESM9_ESM.zip › Figure 6/6C/Fig 6C in-vivo-co-ip-ip-flag-myc.tif]
