# Supplementary material for: The Mediator kinase module enhances polymerase activity to regulate transcriptional memory after heat stress in Arabidopsis
Source: EMBO J. 2024 Jan 16;43(3):6. doi: 10.1038/s44318-023-00024-x (PMC10897291; doi:10.1038/s44318-023-00024-x)
Supplement: Supplementary file 11 — Expanded View Figures [file 44318_2023_24_MOESM11_ESM.pdf]

Expanded View Figures

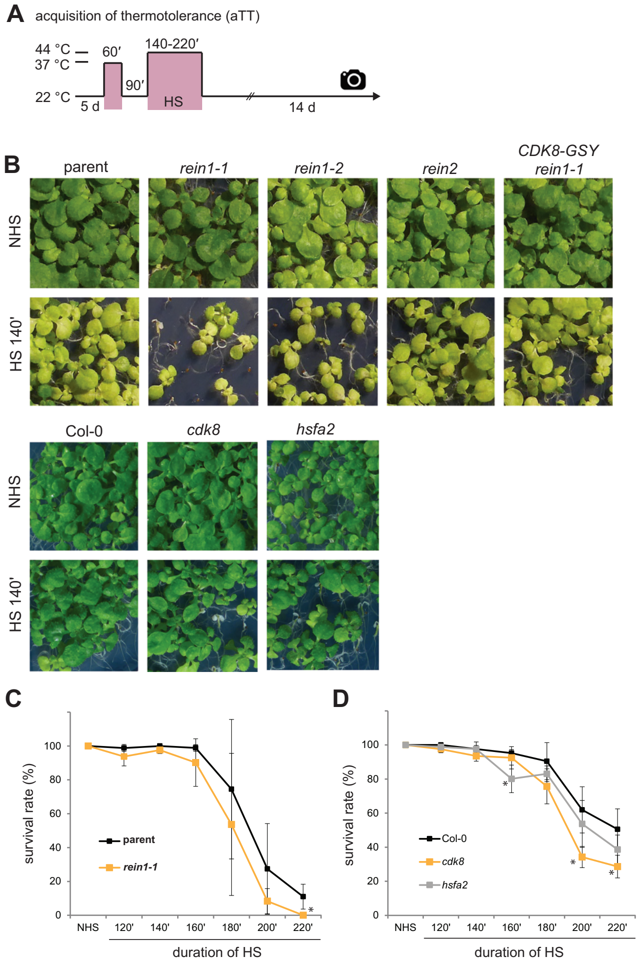

Figure EV1. Acquisition of thermotolerance is slightly impaired in *rein* and *cdk8*.

(**A**) Treatment scheme for acquired thermotolerance (aTT) assay. 5 d-old seedlings were exposed to 37 °C for 1 h, recovered at 23 °C for 90 min and subsequently exposed to 44 °C for 140–220 min. Images were taken 14 d later. Three or four biological repeat experiments were performed. (**B**) Representative aTT assay with the *rein1-1, rein1-2* and *rein2* mutants, the complementing *CDK8-GSY rein1-1* line and the parental *pAPX2::LUC* line (upper), and the *cdk8* and *hsfa2* mutants and their parental Col-0 wild type (lower). NHS (non-heat stressed) seedlings are shown as controls for normal growth. (**C**, **D**) Survival rates for *rein1-1* (**C**) and *cdk8, hsfa2* (**D**). Error bars indicate the mean ± SEM of three independent biological replicate experiments. Asterisks denote significant difference of the mutant to the relative control (*$p < 0.05$; unpaired, two-tailed *t* test).

© The Author(s)

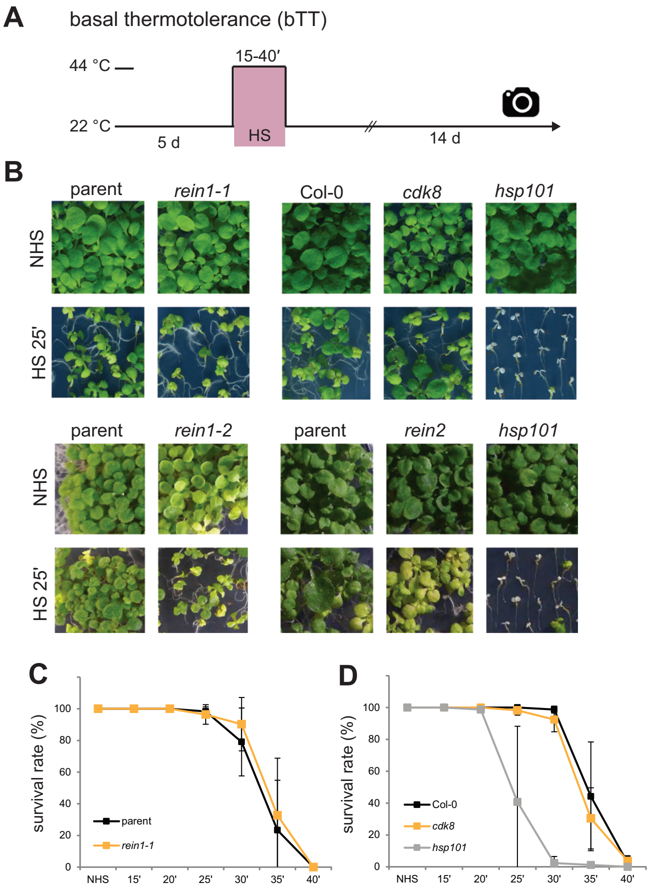

Figure EV2. Basal thermotolerance is not affected in *rein* and *cdk8*.

(**A**) Treatment scheme for basal thermotolerance (bTT) assays. Five-day-old seedlings were exposed to 44 °C for 15-40 min and images were taken 14 d later. The *hsp101* mutant was included as a control for decreased basal thermotolerance. Three or four biological repeat experiments were performed. (**B**) Representative bTT assay with *rein1-1* and its parent line (upper left), *cdk8*, *hsp101* and Col-0 wild type (upper right), *rein1-2* and the parent (lower left), and *rein2* (lower right). Each panel shows representative images from plants grown on the same plates. (**C**, **D**) Survival rates for *rein1-1* (**C**) and *cdk8* (**D**) versus their respective controls Error bars indicate the mean ± SEM of three independent biological replicate experiments.

© The Author(s)

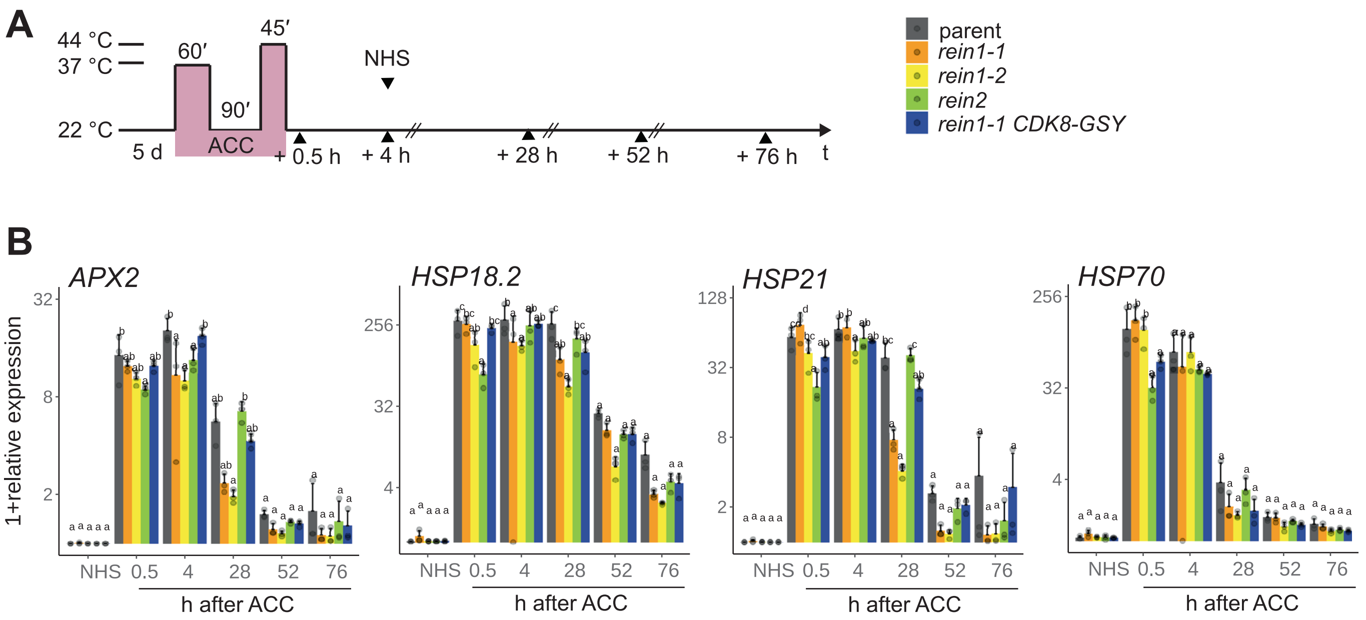

Figure EV3. Type I transcriptional HS memory gene expression is impaired in *rein* mutants.

(**A**) Treatment schema for the type I HS memory assay (sustained induction). Seedlings were grown for 5 d before being subjected to either a full ACC (37 °C for 60 min, RT for 90 min, 44 °C for 45 min). Seedlings were left to recover and sampled at 0.5, 4, 28, 52 or 76 h after the end of ACC (or 4 h after NHS). (**B**) Seedlings were treated as indicated and relative transcript levels of three type I memory genes (*APX2*, *HSP18.2*, *HSP21*) and the HS-induced non-memory gene *HSP70* were measured by qRT-PCR and normalized to the expression of *At4g26410*. Data are $\log_2$(1+mean of relative transcript expression) ± SEM of three independent biological replicate experiments, along with individual data points. Transcript levels were statistically evaluated for all genotypes within each timepoint by ANOVA followed by Tukey's HSD test ($p < 0.05$). Genotypes are assigned one or more letters based on their statistical group. Genotypes sharing one letter are not significantly different.

 © The Author(s)

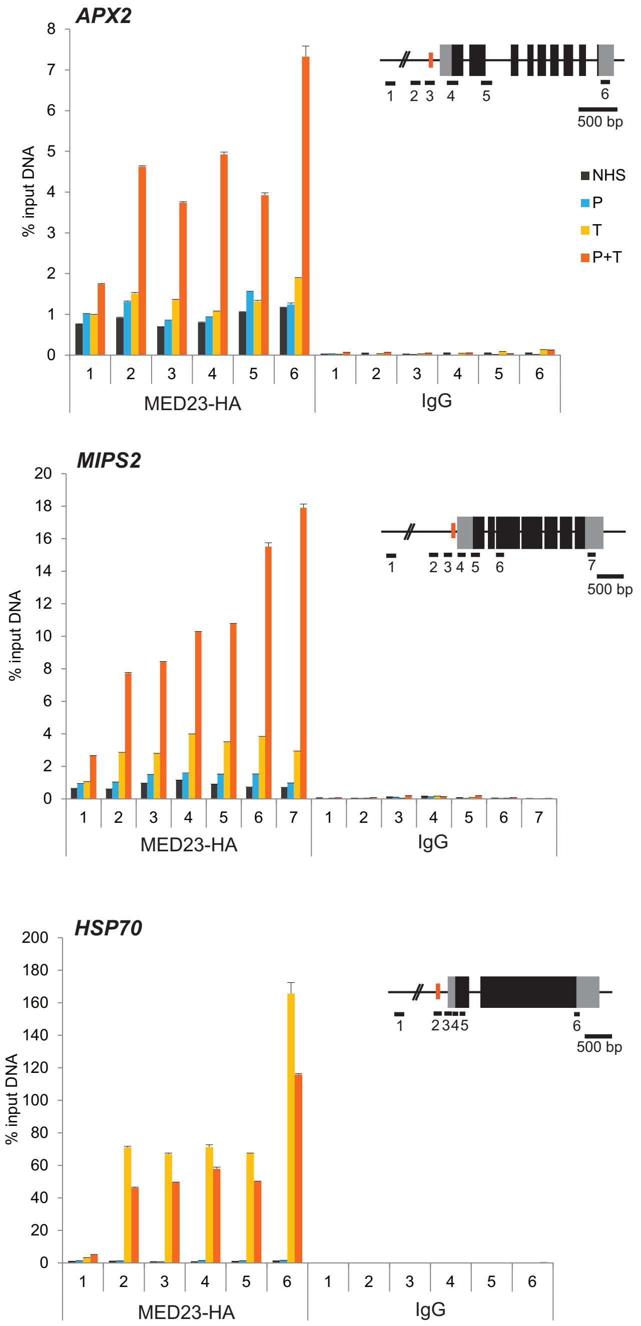

Figure EV4. MED23 is recruited to HS memory gene loci during type II memory.

Seedlings of the MED23-HA line were subjected to a type II HS regime. Occupancy of MED23-HA was determined by ChIP-qPCR using antibodies against the HA tag. Amplicons targeted the sites indicated on the gene models; red bars indicate binding sites for HSFA2. Data from one representative biological replicate are shown ±SEM of three technical replicates; the experiment was repeated with near identical results.

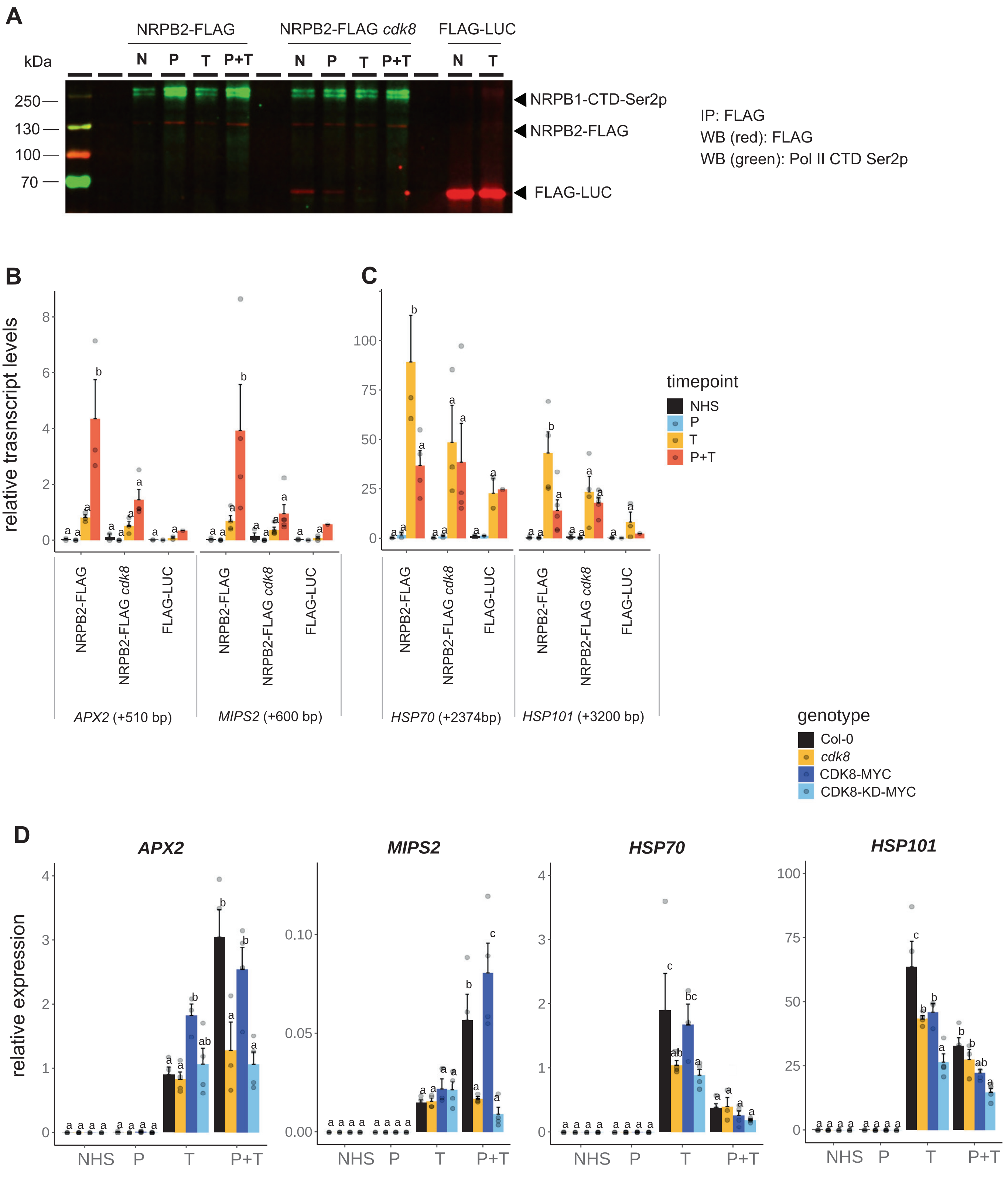
A
NRPB2-FLAG
NRPB2-FLAG cdk8
FLAG-LUC
kDa
N P T P+T N P T P+T N T
250
130
100
70
NRPB1-CTD-Ser2p
NRPB2-FLAG
FLAG-LUC
IP: FLAG
WB (red): FLAG
WB (green): Pol II CTD Ser2p
B
relative trasnscript levels
APX2 (+510 bp)
MIPS2 (+600 bp)
C
HSP70 (+2374bp)
HSP101 (+3200 bp)
timepoint
NHS
P
T
P+T
genotype
Col-0
cdk8
CDK8-MYC
CDK8-KD-MYC
D
APX2
MIPS2
HSP70
HSP101
relative expression
NHS P T P+T

 © The Author(s)

Figure EV5. Pol II-associated nascent transcript levels of type II memory gene loci depend on CDK8.

NRPB2-FLAG seedlings in the wild type or *cdk8* background, and FLAG-LUC control seedlings, were subjected to a type II HS regime (cf. Fig. 4A). NHS, no heat stress; P, HS on d 5 only (primed); T, HS on d 7 only (triggered); P + T, HS on both d 5 and d 7 (primed + triggered); each HS consisted of 37 °C for 60 min, and seedlings were sampled on d 7. (**A**) Native RNA polymerase II complexes were immunoprecipitated from total protein extracts and separated by SDS-PAGE, followed by immunoblotting with antibodies against FLAG (red) or RNA pol II (NRPB1-)CTD-Ser2p (green). (**B**, **C**) Nascent elongating transcript levels in isolated native RNA polymerase II complexes were analysed using plaNET-qPCR. (**D**) Seedlings of Col-0, *cdk8*, CDK8-MYC or CDK8-KD-MYC were subjected to a type II HS regime and relative levels of the unspliced transcripts of *APX2, MIPS2, HSP70* and *HSP101* were measured by qRT-PCR. (**B**, **D**) Data shown are the mean ± SEM of indicated transcript levels, normalized to ACTIN2 (**B**, **C**) or *At4g26410* (**D**), as well as individual data points from at least four independent biological repeat experiments, along with individual data points. Transcript levels were statistically evaluated for all genotypes within each treatment by ANOVA followed by Tukey's HSD test ($p < 0.05$). Genotypes are assigned one or more letters based on their statistical group. Genotypes sharing one letter are not significantly different.

© The Author(s)
